# Tissue and serum microRNA profile of oral squamous cell carcinoma patients

**DOI:** 10.1038/s41598-017-18945-z

**Published:** 2018-01-12

**Authors:** Augusto Schneider, Berta Victoria, Yury Nunez Lopez, Wiktoria Suchorska, Wojciech Barczak, Agnieszka Sobecka, Wojciech Golusinski, Michal M. Masternak, Pawel Golusinski

**Affiliations:** 1 Faculdade de Nutrição, Universidade Federal de Pelotas, Pelotas, RS Brazil; 2 College of Medicine, Burnett School of Biomedical Sciences, University of Central Florida, Orlando, Florida 32827 USA; 3 Translational Research Institute for Metabolism and Diabetes, Florida Hospital, Orlando, FL USA; 4 Radiobiology Lab, Department of Medical Physics, Poznan University of Medical Sciences, The Greater Poland Cancer Centre, Poznan, Poland; 5 Department of Head and Neck Surgery, Poznan University of Medical Sciences, The Greater Poland Cancer Centre, Poznan, Poland; 6 Department of Biology and Environmental Studies, Poznan University of Medical Sciences, Poznan, Poland

## Abstract

Head and neck cancer is characterized by malignant tumors arising from the epithelium covering the upper aerodigestive tract, and the majority of these epithelial malignancies are squamous cell carcinomas (SCCs) of the oral cavity (OSCCs). The aim of the current work was to identify miRNAs regulated in OSCC cancerous tissue when compared to a healthy adjacent tissue and to verify the presence of the same miRNAs in the circulation of these patients. For that serum samples and biopsies of healthy and tumor tissues were collected from five patients diagnosed with OSCC of the oral cavity, RNA was extracted from these samples and microRNAs libraries were prepared and sequenced. A total 255 miRNAs were identified in tissue and 381 different miRNAs were identified in serum samples. When comparing the miRNA expression between tumor and healthy tissue we identified 48 miRNAs (25 down- and 23 up-regulated) that were differentially expressed (FDR < 0.05). From these 48 differentially expressed miRNAs in tissue, 30 miRNAs were also found in the serum of the same patients. hsa-miR-32-5p was up-regulated in tumor compared to healthy tissue in our study, and was previously shown to be up-regulated in the serum of OSCC patients. Therefore, this suggests that miRNAs can be used as potential non-invasive biomarkers of OSCC.

## Introduction

Head and neck cancer is characterized by malignant tumors arising from the epithelium covering the upper aerodigestive tract that can metastasize to other organs^1^. The majority of these epithelial malignancies are squamous cell carcinomas (SCCs) of the oral cavity (OSCCs)^1^, occurring mostly in elderly patients 50–70 years old^2^, with approximattely 650,000 new cases per year^3^. The major risk factors for development of OSCC are tobacco and alcohol consumption^4^. Despite the declining use of tobacco products, the incidence of OSCC has increased due to the rise of the prevalence of oncogenic HPV infections, proved to be a causative factor for a subset of OSCC^5,6^, which account for approximately 50% of all OSCC cases in some regions of the world^7^. Therefore, there is still need to better understand the biology and uncover novel biomarkers/predictors of OSCC in order to improve response to therapy^8^.

MicroRNAs (miRNAs) are well known for their roles in cell growth and proliferation, regulating pathways which are critical to cancer development^9^. MicroRNAs are short small non-coding RNAs (sncRNAs) of about 22 nucleotides in length that regulate key cellular processes at the level of mRNA transcription and stability^10^. The RNA molecules first interact with Argonaute proteins that then combine with other proteins in the RNA Induced Silencing Complex (RISC). The RISC binds to the 3′ untranslated region (UTR) of target mRNAs cleaving the transcript and blocking further translation^11^. Increasing evidence has emerged suggesting that miRNAs do not have an exclusively cell autonomous role but can be found in several body fluids, including in the serum^12^. The origin of extracellular miRNAs has not been fully elucidated, but some suggest that passive leakage from damaged cells, and active cell secretion within exosomes or bound to proteins, are possible mechanisms^13^. It is proposed that secreted miRNAs can have a role in intercellular communication, where donor cells can affect gene expression in distant or adjacent target cells. This suggests that serum miRNAs can act either in a hormone-like pattern or even as biomarkers for a variety of pathological conditions^12^.

Since increasing evidence suggests that miRNAs have an active role in cell function, some miRNAs have been shown to be directly involved in oncogenesis, acting as tumor suppressors (e.g. miR-16) or even oncogenes (e.g. miR-17-92 cluster)^14,15^. In this sense, the pattern of locally produced miRNAs in solid tumors is a very important predictor of malignancy and of the response to chemotherapy^9,14^. Additionally, the pattern of circulating miRNAs has been proposed as a marker for several types of cancer with high success and repeatability^12,16,17^, including for the diagnosis of OSCC cases, which we reported previously^18^. Based on these evidences, the aim of the current work was to identify miRNAs regulated in OSCC cancerous tissue when compared to a healthy adjacent tissue and to verify the presence of the same miRNAs in the circulation of these patients.

## Results

**Figure 1:**
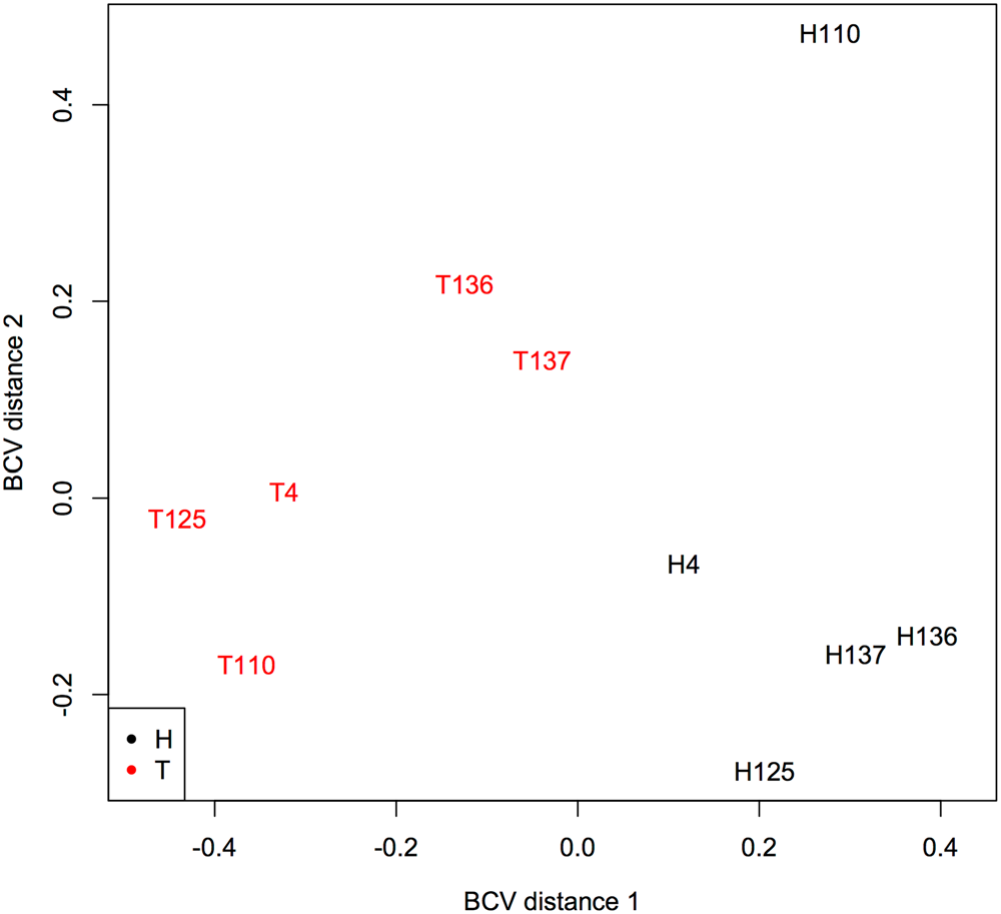
Principal component analysis of the 500 most variable miRNAs in the tissue samples (healthy tissue - H and tumor tissue - T) from five patients diagnosed with HNSCC.

After sequencing and processing of serum and tissue samples (healthy and cancerous paired) from five patients diagnosed with OSCC we obtained an average of 6,391,911 reads/sample with a 67% alignment rate to the human genome (hg19) for serum samples. For tissue samples (healthy and cancerous), an average of 15,594,960 reads/sample was obtained with a 68% alignment rate to the human genome (hg19). Principal component analysis of the 500 most variable miRNAs in the tissue samples (healthy and tumor tissue) indicates a different and very clear pattern of expression between both groups of samples (Fig. 1), indicating the efficiency of the sampling method and sequencing procedures for detecting difference between the two groups of tissues.

After analysis and removal of miRNAs with very low number of reads (<3 reads per million – rpm in less than 50% of the samples) a total 255 miRNAs were identified in tissue (Suppl. Table 1; healthy and tumor paired) and 381 different miRNAs were identified in serum samples (Suppl. Table 2). Of these miRNAs, we were able to identify 214 common miRNAs found both in the tissue and serum of the same patients. When comparing the miRNA expression between tumor and healthy tissue we identified 48 miRNAs (25 down- and 23 up-regulated) that were differentially expressed (False Discovery rate – FDR <0.05 and Fold Change – FC lower than 0.5 or higher than 2.0) (Table 1). From these 48 differentially expressed miRNAs in tissue, 30 miRNAs were also found in the serum of the same patients (Suppl. Table 3).

**Table 1:** MicroRNAs differentially expressed between tumor and healthy adjacent tissue in patients diagnosed with HNSCC.

miRNA	Healthy^1^	Tumor^1^	FC^2^	P Value	FDR^3^
**Down-regulated**					
hsa-miR-204-5p	344.22 ± 91.11	17.55 ± 4	0.06	3.51E-07	1.28E-05
hsa-miR-375	8,515.05 ± 3731.04	1,026.33 ± 475.78	0.09	1.18E-08	1.50E-06
hsa-miR-4497	163.05 ± 43.63	38.93 ± 25.6	0.11	1.51E-05	0.0003
hsa-miR-1291	25.55 ± 6.65	4.97 ± 2.86	0.12	0.0006	0.0048
hsa-miR-4492	2,079.89 ± 449.49	623.76 ± 345.65	0.14	0.0004	0.0034
hsa-miR-3196	52.91 ± 11.59	15.47 ± 8.59	0.14	0.0015	0.0098
hsa-miR-6087	863.11 ± 228.33	197.8 ± 98.4	0.14	3.84E-05	0.0005
hsa-miR-4508	513.24 ± 82.05	165.24 ± 87.58	0.15	1.26E-04	0.0015
hsa-miR-4485-3p	1,123.14 ± 122.84	342.32 ± 174.79	0.16	5.28E-05	0.0006
hsa-miR-3195	169.22 ± 26.06	56.78 ± 31.03	0.16	0.0004	0.0034
hsa-miR-3687	132.19 ± 33.34	43.88 ± 27.87	0.17	0.0009	0.0067
hsa-miR-3648	43.98 ± 11.65	12.65 ± 6.93	0.17	0.0011	0.0079
hsa-miR-6510-3p	163.94 ± 74.97	20.35 ± 4.35	0.17	0.0007	0.0054
hsa-miR-4516	1635 ± 411.42	504.11 ± 268.88	0.17	0.0006	0.0048
hsa-miR-7704	660.64 ± 175.7	219.49 ± 129.52	0.18	0.0003	0.0033
hsa-miR-3656	622.23 ± 139.7	202.24 ± 92.26	0.21	0.0020	0.0122
hsa-let-7c-5p	4,812.94 ± 1102.21	1,129.95 ± 257.38	0.23	1.38E-07	1.17E-05
hsa-miR-4532	5,351.31 ± 955.34	1,714.59 ± 702	0.23	3.93E-05	0.0005
hsa-miR-4488	489.12 ± 102.88	187.6 ± 85.58	0.25	0.0056	0.0305
hsa-miR-99a-3p	20.09 ± 2.83	5.35 ± 1.5	0.25	0.0053	0.0296
hsa-miR-125b-5p	2,016.76 ± 257.48	619.77 ± 211.97	0.26	3.57E-05	0.0005
hsa-miR-139-5p	77.3 ± 10.43	21.21 ± 4.97	0.26	1.89E-05	0.0003
hsa-miR-3651	37.03 ± 7.24	12.6 ± 5.58	0.26	0.0068	0.0362
hsa-miR-125b-2-3p	632.01 ± 120.54	169.59 ± 28.9	0.27	3.77E-06	8.73E-05
hsa-miR-99a-5p	1,185.4 ± 41.23	395.01 ± 93.85	0.30	3.26E-05	0.0005
**Up-regulated**					
hsa-miR-31-3p	1.16 ± 0.98	39.8 ± 7.41	77.11	2.91E-06	8.73E-05
hsa-miR-424-5p	2.14 ± 1.86	39.75 ± 7.37	71.59	9.64E-06	2.05E-04
hsa-miR-196b-5p	2.02 ± 1.75	30.15 ± 10.01	45.71	3.89E-05	0.0005
hsa-miR-877-5p	1.27 ± 1.09	18.72 ± 5.12	35.80	0.0003	0.0031
hsa-miR-7-5p	1.72 ± 1.56	12.84 ± 3.27	19.87	0.0004	0.0034
hsa-miR-135b-5p	4.14 ± 2.98	44.97 ± 7.22	17.78	2.62E-07	1.20E-05
hsa-miR-31-5p	128.27 ± 21.82	2,528.87 ± 1130.27	14.86	1.24E-10	3.17E-08
hsa-miR-142-3p	12.2 ± 6.43	94.3 ± 26.39	11.70	2.82E-07	1.20E-05
hsa-miR-187-3p	3.52 ± 1.93	25.4 ± 6.59	8.44	0.0003	0.0031
hsa-miR-19a-3p	6.74 ± 3.3	33.87 ± 10.76	7.43	0.0018	0.0114
hsa-miR-708-3p	4.06 ± 1.25	25.92 ± 3.89	6.34	3.45E-06	8.73E-05
hsa-miR-223-3p	63.71 ± 13.03	442.93 ± 139.65	6.16	2.24E-07	1.20E-05
hsa-miR-32-5p	5.49 ± 2.43	24.26 ± 6.04	4.91	0.0006	0.0046
hsa-miR-18a-5p	24.29 ± 5.7	123.25 ± 36.61	4.84	3.66E-06	8.73E-05
hsa-miR-301a-3p	9.17 ± 2.54	46.43 ± 12.17	4.52	0.0002	0.0021
hsa-let-7a-3p	23.56 ± 16.17	52.02 ± 10.75	4.32	0.0027	0.0164
hsa-miR-21-5p	11,577 ± 2360.39	40,506.05 ± 5524.39	3.56	2.58E-05	0.0004
hsa-miR-455-5p	18.77 ± 2.14	61.45 ± 8.19	3.12	0.0004	0.0034
hsa-miR-92b-3p	497.22 ± 94.34	1465.13 ± 210.46	3.02	0.0004	0.0034
hsa-miR-21-3p	1,366.33 ± 343.19	3,663.11 ± 527.37	2.90	0.0016	0.0107
hsa-miR-142-5p	816.58 ± 219.92	2,412.08 ± 695.48	2.64	0.0043	0.0254
hsa-miR-944	58.54 ± 12.98	146.36 ± 19.26	2.56	0.0053	0.0296
hsa-miR-20a-5p	87.07 ± 12.5	214.73 ± 26.33	2.41	0.0044	0.0257

^1^miRNAs are expressed as reads per million (rpm). miRNA with less than 3 rpm in less than 50% of the samples were removed from analysis.

^2^Fold change in Tumor compared to Healthy tissue.

^3^False discovery rate. Only miRNAs with FDR lower than 0.05 were considered as significantly regulated.

Pathway and Gene Ontology (GO) term enrichment analysis indicated that several important processes are regulated by these 48 differentially expressed miRNAs, with cancer related pathways among the top 20 regulated pathways (Tables 2 and 3). A full list of regulated pathways is presented in Suppl. Table 4.

**Table 2:** Top 20 pathways of target genes from the 48 miRNAs differentially expressed between tumor and healthy tissue of HNSCC patients.

KEGG pathway	P value^1^	Genes^2^	miRNAs^3^
Hippo signaling pathway	8.98E-09	102	33
Signaling pathways regulating pluripotency of stem cells	2.18E-08	96	34
Fatty acid biosynthesis	8.41E-07	7	18
Axon guidance	1.16E-06	84	31
Proteoglycans in cancer	2.08E-06	126	34
Pathways in cancer	8.41E-06	234	41
TGF-beta signaling pathway	8.56E-06	52	30
Endocytosis	8.56E-06	129	38
Glutamatergic synapse	9.06E-06	72	33
Glioma	1.11E-05	45	31
Neurotrophin signaling pathway	1.11E-05	84	33
Pancreatic cancer	1.46E-05	48	27
Wnt signaling pathway	1.46E-05	91	33
Thyroid hormone signaling pathway	2.14E-05	76	33
Morphine addiction	2.41E-05	58	32
Ras signaling pathway	2.82E-05	137	38
mTOR signaling pathway	4.85E-05	46	29
Renal cell carcinoma	4.93E-05	49	29
ErbB signaling pathway	5.78E-05	62	33
Oxytocin signaling pathway	6.22E-05	100	31

^1^Only pathways with P values lower than 0.05 were considered as significant.

^2^Number of genes affected in the pathway by the regulated miRNAs.

^3^Number of miRNAs differentially expressed that have a target gene in the pathway.

**Table 3:** Top 20 Gene Ontology Terms for biological processes of target genes from the 48 miRNAs differentially expressed in healthy and cancerous tissue of HNSCC patients.

Go category	P value^1^	Genes^2^	miRNAs^3^
Cellular nitrogen compound metabolic process	9.97E-41	2209	29
Cellular protein modification process	2.79E-29	1138	26
Neurotrophin trk receptor signaling pathway	1.13E-13	178	25
Biosynthetic process	1.94E-28	1818	25
Gene expression	2.61E-11	293	19
Fc-epsilon receptor signaling pathway	6.53E-10	94	18
Epidermal growth factor receptor signaling pathway	1.36E-08	116	15
Biological_process	4.23E-09	5271	14
Catabolic process	8.47E-08	730	14
Phosphatidylinositol-mediated signaling	6.84E-06	66	13
Small molecule metabolic process	1.84E-10	782	12
Fibroblast growth factor receptor signaling pathway	1.22E-06	92	11
Cellular component assembly	4.42E-07	437	11
Symbiosis, encompassing mutualism through parasitism	1.52E-08	218	11
Viral process	8.65E-08	197	11
Cell-cell signaling	0.00022	213	10
Synaptic transmission	8.52E-05	170	10
Transcription, dna-templated	4.75E-08	703	10
Response to stress	7.46E-06	664	9
Blood coagulation	1.82E-07	166	9
Transcription initiation from rna polymerase ii promoter	0.0011	82	8
Macromolecular complex assembly	1.42E-05	249	7
Cellular lipid metabolic process	6.74E-06	58	7
Nervous system development	2.30E-05	98	6
Nucleobase-containing compound catabolic process	0.00023	248	6
Cell death	0.0003	256	6

^1^Only GO Terms with P values lower than 0.05 were considered as significant.

^2^Number of genes affected in the process by the regulated miRNAs.

^3^Number of miRNAs differentially expressed that have a target gene in the process.

Additionally, we performed a correlation analysis between the fold change expression between healthy/cancer tissue and serum of these 48 miRNAs, which indicated no significant association between its expression in serum and tissue (all P > 0.05).

## Discussion

In the current study, we were able to identify a characteristic signature of miRNA expression in cancerous tissue samples from OSCC patients. Several of the miRNAs regulated in OSCC cancerous tissue were also found in the circulation and can be used as potential biomarkers. In a review by Volinia, *et al*.^9^, it was identified 21 miRNAs that are commonly expressed in solid lung, breast, stomach, prostate, colon, and pancreatic tumors. Of these 21 miRNAs identified by the authors, we observed that four miRNAs were also differentially regulated between healthy and tumor tissue in our OSCC patients, namely hsa-miR-21, hsa-miR-20a, hsa-miR-223 and hsa-miR-32. Another review profiling the miRNAs aberrant expressed specifically in OSCC patients also identified hsa-miR-21 and hsa-miR-223 as important markers, although hsa-miR-20a and hsa-miR-32 were not^19^. Additionally, hsa-miR-375 and hsa-miR-31 were previously identified as changed in OSCC^19^, which is in agreement with our current observations as the top changed miRNAs.

hsa-miR-21-5p was one of the highest expressed miRNAs, and was up-regulated in cancerous compared to healthy tissue in our study. A previous study also identified hsa-miR-21-5p as up-regulated in OSCC patients cancerous tissue compared to healthy adjacent tissue^20^. This miRNA is considered an oncogene and is the most commonly over-expressed miRNA in several cancerous tissues^9^. Overexpression of hsa-miR-21 was reported to be inversely correlated with drug sensitivity in chemoterapy and progression-free survival^21^. miR-21 is known for regulating cell growth and proliferation by targeting PTEN, and therefore its over-expression is associated with the activation of the Pi3k/Akt pathway and rapid cell growth^22^. This findings suggest hsa-miR-21 as an important biomarker of survival and response to treatment, as well as a key player in the malignancy development in OSCC.

hsa-miR-375 was also one the highest expressed miRNAs in tissue samples and it was almost 10 times less expressed in cancerous tissue compared to healthy adjacent tissue. In addition, the change in expression of hsa-miR-375 between healthy and cancer tissue was significantly correlated with the expression of this miRNA in serum. Others have also previously observed down-regulation of hsa-miR-375 in cancer tissue of OSCC patients^23^. A previous study also identified that the expression levels of hsa-miR-21/ hsa-miR-375 were a good predictor of prognosis and its ratio was higher in later stages laryngeal SCC^24^. hsa-miR-375 was observed to be down-regulated in serum of patients diagnosed with oropharyngeal SCC, and highly associated with cancer recurrence^25^. The levels of hsa-miR-375 regulates the expression of MMP13, therefore promoting increased metastatic behavior and cancer aggressiveness in esophageal SCC^26^.

hsa-miR-31-3p was another one of the highest expressed miRNAs in tissue and was almost 20 times more expressed in cancerous than healthy adjacent tissue. hsa-miR-31 is considered an oncogene and was previously associated with decreased survival, associated to increased Nanog/OCT4/Sox2/EpCAM expression and stemness of cancerous tissue^27^. A previous study evaluating oral and pharyngeal SCC patients also identified hsa-miR-31 as the most up-regulated miRNA^28^. The same study also identified hsa-miR-375 as the most down-regulated miRNA, which is in alignment with our current observations, further corroborating with the idea that a cancer specific signature of miRNAs can be established and used for diagnostic purposes. hsa-miR-31 was also shown to be an important regulator of tumor suppressor genes, and its knockdown results in decreased cell proliferation and tumorigenicity in lung cancer^29^.

A previous case-control study from our group identified a serum miRNA signature expression profile characteristic of patients diagnosed with OSCC^18^. When we overlap these 28 serum regulated miRNAs with the ones we identified as differentially regulated between tumorous and healthy adjacent tissue of OSCC patients of the current study we identified that hsa-miR-99a-5p, was down-regulated in tumor tissue and up-regulated in serum of tumor patients, and hsa-let-7a-3p was up-regulated in tumor tissue and down-regulated in serum of tumor patients, while hsa-miR-32-5p, was up-regulated in tumor tissue and in serum of tumor patients. Therefore, these miRNAs can be good candidates as markers for non-invasive diagnosis of patients with OSCC, in particular hsa-miR-32-5p, since it was up-regulated in the tissue and in serum of OSCC patients. As mentioned before, previous studies identified hsa-miR-32 as a good candidate miRNA in a myriad of solid cancers^9^, but its presence also in serum indicates that non-invasive diagnostic methods can also be applied and further validated in larger groups of patients.

As discussed before, several of the miRNAs differentially expressed are known to be directly involved in cancer development and progression, therefore it is no surprise that several pathways related to cancer in different types of tissue were among the most significantly enriched pathways. Additionally, Proteoglycans, p53 signaling pathway and pathways in cancer were also among the most regulated pathways.

To sum up, we identified 48 miRNAs differentially expressed in cancerous compared to healthy tissue in OSCC patients and many of these miRNAs are also found in serum of the same patients. Therefore, this suggests that miRNAs can be used as potential non-invasive biomarkers of OSCC progression during follow-up as well as survival marker in future studies. This study highlights the importance of hsa-miR-32-5p, which is up-regulated in cancerous OSCC tissue and was previously reported to be up-regulated in serum of OSCC patients^18^.

## Methods

### Patients

The clinical material comprised the tumor tissue from five patients (2 males and 3 females, 64.4 ± 5.6 years old) diagnosed with squamous cell carcinoma of the oral cavity at Department of Head and Neck Surgery at The Greater Poland Cancer Centre in Poznan, Poland. All patients have been qualified by the institutional Multidisciplinary Team for the primary surgical resection. Recurrences and patients initially treated with other therapeutic modalities were not included in the study. The patients with the HPV positive malignancies were also excluded from the study. All tumors were confirmed by the pathologist and have been collected intraoperatively as it was previously completed for our preliminary experiments and already published data^30,31^. OSCC tumors have be staged based on TNM classification of Malignant Tumors, which describes in detail the extent of subject’s cancer^32^. All clinical material has been classified based on the guidelines from the International Union Against Cancer using standard TNM classification. Additionally, pathological TNM classification for each patient at the Greater Poland Cancer Center was performed. All of evaluated cases were pathologically confirmed squamous cell carcinoma. Three of the five cases were located at the floor of the mouth and remaining two in the body of the tongue. All cases has been described on histopathological analysis as pT2 N1 M0.

### Sample and tissue collection

Blood samples were collected from the patients, prior to any surgical intervention and centrifuged for serum separation. From every patient two separate tissue specimens were obtained during surgical resection. Core biopsy from the tumor and healthy mucosa were collected to allow comparison of tumor site versus non-tumor healthy tissue, in the same cancer patient. Specimens were immediately frozen in liquid nitrogen and then stored in −80 °C.

### RNA extraction and miRNA library preparation

The tissues samples (n = 10) were removed from the −80 °C freezer and homogenized with Qiazol (Qiagen, Valencia, CA, USA) using 0.5 mm zirconium oxide beads in the Bullet Blender 24 (Next Advance, Averill Park, NY, USA). Total RNA was extracted using a commercial column purification system (miRNeasy Mini Kit, Qiagen) and on-column DNase treatment (RNase-free DNase Set, Qiagen) following manufacturer’s instructions. Serum samples (n = 5) were extracted using the miRNEasy Serum/Plasma kit (Qiagen) following manufactures instructions, but adjusting for an initial volume of 300 uL of serum.

MicroRNA libraries were prepared using the TruSeq Small RNA Sample Preparation Kit (Illumina Inc., San Diego, CA, USA) following the manufacturer’s instructions and adjusted by Matkovich, *et al*.^33^. Briefly, small RNAs from 1 μg of total RNA or from 300 uL of serum (not quantified) were ligated with 3′ and 5′ adapters, followed by reverse transcription to produce single stranded cDNAs. Samples were then amplified by PCR in 14 cycles (94 °C for 30 sec, 14 cycles of 94 °C for 15 sec, 62 °C for 30 sec and 70 °C for 15 sec, and a final extension of 70 °C for 5 min) using indexes to allow all individual libraries to be processed in a single flowcell lane during sequencing. The amplified libraries were size-selected and purified in a 6% agarose gel.

The quantity and quality of miRNA libraries was determined using BioAnalyzer and RNA Nano Lab Chip Kit (Agilent Technologies, Santa Clara, CA, USA), and the samples were combined in a single microtube and submitted to sequencing on a HiSeq. 2500 instrument (Illumina Inc.) using the Illumina HiSeq v4 kit in a single read 50 bp (1 × 50) run.

### miRNAs libraries analysis and statistical analyses

The alignment of sequences from the libraries obtained in the previous step and the quantification of miRNAs was performed using the sRNAtoolbox webserver according to Rueda *et al*.^34^. Statistical analyses of differentially expressed miRNAs was performed using EdgeR^35^ using the GLM model and pairing samples by patient. miRNAs with a FDR < 0.05 and FC > 2.0 were considered as up-regulated; and FDR < 0.05 and FC < 0.50 were considered as down-regulated. Unsupervised hierarchical clustering for the 50 most expressed miRNAs was performed using the software R (3.2.2) and the Bioconductor package DESeq (1.2.0)^36^. miRNA gene families were identified using miRBase^37^.

### miRNAs target prediction and enriched pathways and GO Terms

The mirPath tool (version 3.0) was used to predict target genes of the differentially regulated miRNAs using the microT-CDS v. 5.0 database^38^. The same tool was used for retrieving gene ontology (GO) terms (biological processes) and KEGG molecular pathways^39,40^ having at least 5 miRNAs targeting the process or pathway. P values lower than 0.05 were considered as significant.

#### Statement

All authors declare, that all methods in this study followed the protocol approved by the Institutional Review Board of Poznan University of Medical Sciences in Poznan, Poland. All experiments were performed in accordance with relevant guidelines and regulations. Informed consent for participation in the study has been obtained from all patients included in the study.

## Electronic supplementary material

Supplementary Tables
