# Supplementary material for: Tissue and serum microRNA profile of oral squamous cell carcinoma patients
Source: Sci Rep. 2018 Jan 12;8:675. doi: 10.1038/s41598-017-18945-z (PMC5766573; doi:10.1038/s41598-017-18945-z)
Supplement: Supplementary file 1 — Supplementary Tables [file 41598_2017_18945_MOESM1_ESM.doc]

**Tissue and serum microRNA profile of oral squamous cell carcinoma patients**

Augusto Schneider^1,2^, Berta Victoria^2^, Yury Nunez Lopez^3^, Wiktoria Suchorska^4^, Wojciech Barczak^4,5^, Agnieszka Sobecka^4,5^, Wojciech Golusinski^5^, Michal M. Masternak^2,5^, Pawel Golusinski^2,5,6^*

^1^Faculdade de Nutrição, Universidade Federal de Pelotas, Pelotas, RS, Brazil

^2^College of Medicine, Burnett School of Biomedical Sciences, University of Central Florida, Orlando, FL 32827

^3^Translational Research Institute for Metabolism and Diabetes, Florida Hospital, Orlando, FL, USA

^4^Radiobiology Lab, Department of Medical Physics, Poznan University of Medical Sciences, The Greater Poland Cancer Centre, Poznan, Poland

^5^Department of Head and Neck Surgery, Poznan University of Medical Sciences, The Greater Poland Cancer Centre, Poznan, Poland

^6^Department of Biology and Environmental Studies, Poznan University of Medical Sciences, Poznan, Poland

***Corresponding author:**

Pawel Golusinski, M. D., Ph.D.

Department of Head and Neck Surgery

Garbary 15, 61-866 Poznan Poland

pawel.golusinski@wco.pl

Supplemental Table 1 – MicroRNAs expressed in tumor and healthy adjacent tissue in patients diagnosed with HNSCC.

| microRNAs | Healthy | Tumor | FC | P Value | FDR |
| --- | --- | --- | --- | --- | --- |
| hsa-miR-31-5p | 128.27 ± 21.82 | 2528.87 ± 1130.27 | 14.86 | 1.24E-10 | 3.17E-08 |
| hsa-miR-375 | 8515.05 ± 3731.04 | 1026.33 ± 475.78 | 0.09 | 1.18E-08 | 1.50E-06 |
| hsa-let-7c-5p | 4812.94 ± 1102.21 | 1129.95 ± 257.38 | 0.23 | 0.000000138 | 1.17E-05 |
| hsa-miR-223-3p | 63.71 ± 13.03 | 442.93 ± 139.65 | 6.16 | 0.000000224 | 1.20E-05 |
| hsa-miR-135b-5p | 4.14 ± 2.98 | 44.97 ± 7.22 | 17.78 | 0.000000262 | 1.20E-05 |
| hsa-miR-142-3p | 12.2 ± 6.43 | 94.3 ± 26.39 | 11.70 | 0.000000282 | 1.20E-05 |
| hsa-miR-204-5p | 344.22 ± 91.11 | 17.55 ± 4 | 0.06 | 0.000000351 | 1.28E-05 |
| hsa-miR-31-3p | 1.16 ± 0.98 | 39.8 ± 7.41 | 77.11 | 2.91E-06 | 8.73E-05 |
| hsa-miR-708-3p | 4.06 ± 1.25 | 25.92 ± 3.89 | 6.34 | 3.45E-06 | 8.73E-05 |
| hsa-miR-18a-5p | 24.29 ± 5.7 | 123.25 ± 36.61 | 4.84 | 3.66E-06 | 8.73E-05 |
| hsa-miR-125b-2-3p | 632.01 ± 120.54 | 169.59 ± 28.9 | 0.27 | 3.77E-06 | 8.73E-05 |
| hsa-miR-424-5p | 2.14 ± 1.86 | 39.75 ± 7.37 | 71.59 | 9.64E-06 | 2.05E-04 |
| hsa-miR-4497 | 163.05 ± 43.63 | 38.93 ± 25.6 | 0.11 | 1.51E-05 | 0.0003 |
| hsa-miR-139-5p | 77.3 ± 10.43 | 21.21 ± 4.97 | 0.26 | 1.89E-05 | 0.0003 |
| hsa-miR-21-5p | 11577 ± 2360.39 | 40506.05 ± 5524.39 | 3.56 | 2.58E-05 | 0.0004 |
| hsa-miR-99a-5p | 1185.4 ± 41.23 | 395.01 ± 93.85 | 0.30 | 3.26E-05 | 0.0005 |
| hsa-miR-125b-5p | 2016.76 ± 257.48 | 619.77 ± 211.97 | 0.26 | 3.57E-05 | 0.0005 |
| hsa-miR-6087 | 863.11 ± 228.33 | 197.8 ± 98.4 | 0.14 | 3.84E-05 | 0.0005 |
| hsa-miR-196b-5p | 2.02 ± 1.75 | 30.15 ± 10.01 | 45.71 | 3.89E-05 | 0.0005 |
| hsa-miR-4532 | 5351.31 ± 955.34 | 1714.59 ± 702 | 0.23 | 3.93E-05 | 0.0005 |
| hsa-miR-4485-3p | 1123.14 ± 122.84 | 342.32 ± 174.79 | 0.16 | 5.28E-05 | 0.0006 |
| hsa-miR-4508 | 513.24 ± 82.05 | 165.24 ± 87.58 | 0.15 | 1.26E-04 | 0.0015 |
| hsa-miR-301a-3p | 9.17 ± 2.54 | 46.43 ± 12.17 | 4.52 | 0.0002 | 0.0021 |
| hsa-miR-877-5p | 1.27 ± 1.09 | 18.72 ± 5.12 | 35.80 | 0.0003 | 0.0031 |
| hsa-miR-187-3p | 3.52 ± 1.93 | 25.4 ± 6.59 | 8.44 | 0.0003 | 0.0031 |
| hsa-miR-7704 | 660.64 ± 175.7 | 219.49 ± 129.52 | 0.18 | 0.0003 | 0.0033 |
| hsa-miR-92b-3p | 497.22 ± 94.34 | 1465.13 ± 210.46 | 3.02 | 0.0004 | 0.0034 |
| hsa-miR-7-5p | 1.72 ± 1.56 | 12.84 ± 3.27 | 19.87 | 0.0004 | 0.0034 |
| hsa-miR-3195 | 169.22 ± 26.06 | 56.78 ± 31.03 | 0.16 | 0.0004 | 0.0034 |
| hsa-miR-4492 | 2079.89 ± 449.49 | 623.76 ± 345.65 | 0.14 | 0.0004 | 0.0034 |
| hsa-miR-455-5p | 18.77 ± 2.14 | 61.45 ± 8.19 | 3.12 | 0.0004 | 0.0034 |
| hsa-miR-32-5p | 5.49 ± 2.43 | 24.26 ± 6.04 | 4.91 | 0.0006 | 0.0046 |
| hsa-miR-1291 | 25.55 ± 6.65 | 4.97 ± 2.86 | 0.12 | 0.0006 | 0.0048 |
| hsa-miR-4516 | 1635 ± 411.42 | 504.11 ± 268.88 | 0.17 | 0.0006 | 0.0048 |
| hsa-miR-6510-3p | 163.94 ± 74.97 | 20.35 ± 4.35 | 0.17 | 0.0007 | 0.0054 |
| hsa-miR-3687 | 132.19 ± 33.34 | 43.88 ± 27.87 | 0.17 | 0.0009 | 0.0067 |
| hsa-miR-3648 | 43.98 ± 11.65 | 12.65 ± 6.93 | 0.17 | 0.0011 | 0.0079 |
| hsa-miR-3196 | 52.91 ± 11.59 | 15.47 ± 8.59 | 0.14 | 0.0015 | 0.0098 |
| hsa-miR-21-3p | 1366.33 ± 343.19 | 3663.11 ± 527.37 | 2.90 | 0.0016 | 0.0107 |
| hsa-miR-19a-3p | 6.74 ± 3.3 | 33.87 ± 10.76 | 7.43 | 0.0018 | 0.0114 |
| hsa-miR-3656 | 622.23 ± 139.7 | 202.24 ± 92.26 | 0.21 | 0.0020 | 0.0122 |
| hsa-let-7a-3p | 23.56 ± 16.17 | 52.02 ± 10.75 | 4.32 | 0.0027 | 0.0164 |
| hsa-miR-142-5p | 816.58 ± 219.92 | 2412.08 ± 695.48 | 2.64 | 0.0043 | 0.0254 |
| hsa-miR-20a-5p | 87.07 ± 12.5 | 214.73 ± 26.33 | 2.41 | 0.0044 | 0.0257 |
| hsa-miR-99a-3p | 20.09 ± 2.83 | 5.35 ± 1.5 | 0.25 | 0.0053 | 0.0296 |
| hsa-miR-944 | 58.54 ± 12.98 | 146.36 ± 19.26 | 2.56 | 0.0053 | 0.0296 |
| hsa-miR-4488 | 489.12 ± 102.88 | 187.6 ± 85.58 | 0.25 | 0.0056 | 0.0305 |
| hsa-miR-3651 | 37.03 ± 7.24 | 12.6 ± 5.58 | 0.26 | 0.0068 | 0.0362 |
| hsa-miR-429 | 110.81 ± 15.88 | 248.82 ± 57.83 | 2.09 | 0.0122 | 0.0635 |
| hsa-miR-1275 | 5.55 ± 1.65 | 13.58 ± 0.98 | 2.67 | 0.0139 | 0.0711 |
| hsa-miR-27a-5p | 25.85 ± 4.59 | 56.42 ± 7.73 | 2.16 | 0.0149 | 0.0747 |
| hsa-miR-181a-2-3p | 115.82 ± 21.14 | 62.36 ± 17.7 | 0.50 | 0.0154 | 0.0754 |
| hsa-miR-378a-5p | 74.58 ± 8.29 | 34.29 ± 8.99 | 0.41 | 0.0183 | 0.0882 |
| hsa-miR-19b-3p | 134.47 ± 38.41 | 251.3 ± 30.1 | 2.15 | 0.0188 | 0.0888 |
| hsa-miR-378a-3p | 5387.72 ± 1204.77 | 2301.26 ± 313.69 | 0.44 | 0.0194 | 0.0899 |
| hsa-miR-16-2-3p | 14.81 ± 6.79 | 29.12 ± 4.92 | 3.17 | 0.0238 | 0.1086 |
| hsa-miR-33b-5p | 6.75 ± 2.63 | 20.75 ± 8.12 | 2.91 | 0.0252 | 0.1128 |
| hsa-miR-1246 | 1917.43 ± 378.22 | 1012.96 ± 293.62 | 0.44 | 0.0292 | 0.1283 |
| hsa-miR-4449 | 45.16 ± 10.26 | 13.89 ± 6.68 | 0.20 | 0.0326 | 0.1405 |
| hsa-miR-210-5p | 29.63 ± 13.23 | 10.23 ± 1.06 | 0.44 | 0.0335 | 0.1405 |
| hsa-miR-10a-5p | 3365.4 ± 763.43 | 6408.1 ± 1309.86 | 2.12 | 0.0336 | 0.1405 |
| hsa-miR-148a-3p | 7231.67 ± 1472.47 | 13984.98 ± 2330.97 | 1.94 | 0.0416 | 0.1710 |
| hsa-miR-26a-5p | 57035.9 ± 6333.75 | 33798 ± 4959.64 | 0.57 | 0.0464 | 0.1879 |
| hsa-miR-17-5p | 114.7 ± 11.69 | 226.81 ± 43.14 | 1.82 | 0.0484 | 0.1904 |
| hsa-miR-130b-3p | 49.4 ± 5.43 | 90.77 ± 9.87 | 1.78 | 0.0485 | 0.1904 |
| hsa-miR-874-3p | 85.62 ± 19.09 | 39.84 ± 5.36 | 0.50 | 0.0511 | 0.1933 |
| hsa-miR-577 | 9.86 ± 2.82 | 33.78 ± 17.24 | 2.84 | 0.0514 | 0.1933 |
| hsa-let-7i-3p | 19.46 ± 5.19 | 45.87 ± 8.72 | 2.42 | 0.0518 | 0.1933 |
| hsa-miR-378c | 83.77 ± 14.87 | 43.2 ± 9.32 | 0.47 | 0.0523 | 0.1933 |
| hsa-miR-24-2-5p | 25.24 ± 2.22 | 51.3 ± 9.59 | 1.87 | 0.0547 | 0.1994 |
| hsa-miR-10b-5p | 98615.02 ± 14679.01 | 59167.24 ± 5525.14 | 0.61 | 0.0637 | 0.2267 |
| hsa-miR-100-5p | 2766.1 ± 797.2 | 964.96 ± 220.64 | 0.35 | 0.0640 | 0.2267 |
| hsa-miR-574-3p | 441.17 ± 55.48 | 250.79 ± 39.03 | 0.55 | 0.0651 | 0.2275 |
| hsa-miR-328-3p | 33.72 ± 3.57 | 19.36 ± 2.17 | 0.56 | 0.0669 | 0.2304 |
| hsa-miR-3182 | 71.39 ± 21.5 | 53.82 ± 25.91 | 0.50 | 0.0684 | 0.2326 |
| hsa-miR-146b-3p | 27.47 ± 6.75 | 55.29 ± 18.51 | 1.80 | 0.0810 | 0.2718 |
| hsa-miR-99b-3p | 12.85 ± 3.54 | 24.38 ± 4.84 | 2.23 | 0.0860 | 0.2848 |
| hsa-miR-185-5p | 14.05 ± 3.86 | 25 ± 2.09 | 1.87 | 0.0883 | 0.2870 |
| hsa-miR-125a-5p | 4571.64 ± 577.55 | 3087.96 ± 826.52 | 0.61 | 0.0889 | 0.2870 |
| hsa-miR-146b-5p | 4465.96 ± 976.18 | 8502.54 ± 2264.38 | 1.75 | 0.0948 | 0.3015 |
| hsa-miR-454-3p | 40.84 ± 8.38 | 66.98 ± 7.9 | 1.69 | 0.0958 | 0.3015 |
| hsa-miR-200a-3p | 148.29 ± 22.68 | 272.59 ± 62.44 | 1.70 | 0.0976 | 0.3034 |
| hsa-let-7f-1-3p | 9.96 ± 3.52 | 16.4 ± 2.89 | 1.91 | 0.0997 | 0.3063 |
| hsa-miR-452-5p | 24.39 ± 7.64 | 53.63 ± 11.48 | 2.36 | 0.1137 | 0.3417 |
| hsa-miR-29b-3p | 41.83 ± 12.15 | 92.24 ± 23.57 | 2.00 | 0.1139 | 0.3417 |
| hsa-miR-130a-3p | 229.71 ± 38.33 | 388.15 ± 58.15 | 1.67 | 0.1163 | 0.3449 |
| hsa-miR-15a-5p | 124.64 ± 11.28 | 203.76 ± 29.43 | 1.54 | 0.1191 | 0.3491 |
| hsa-miR-708-5p | 72.19 ± 11.38 | 111.85 ± 10.25 | 1.57 | 0.1228 | 0.3543 |
| hsa-miR-320b | 36.87 ± 7.47 | 61.41 ± 10.51 | 1.67 | 0.1237 | 0.3543 |
| hsa-miR-340-5p | 78.64 ± 17.78 | 123.18 ± 8.96 | 1.69 | 0.1352 | 0.3779 |
| hsa-miR-500a-3p | 319.12 ± 89.41 | 181.73 ± 21.92 | 0.62 | 0.1363 | 0.3779 |
| hsa-miR-1260b | 85.85 ± 19.73 | 55.13 ± 17.55 | 0.57 | 0.1376 | 0.3779 |
| hsa-miR-141-5p | 56.05 ± 10.82 | 85.78 ± 8.1 | 1.56 | 0.1392 | 0.3779 |
| hsa-miR-15b-3p | 15.3 ± 3.55 | 27.21 ± 5.25 | 1.79 | 0.1400 | 0.3779 |
| hsa-miR-192-5p | 437.67 ± 36.25 | 762.83 ± 160.4 | 1.57 | 0.1420 | 0.3779 |
| hsa-miR-548av-5p | 8.63 ± 2.21 | 16.22 ± 2.66 | 1.99 | 0.1437 | 0.3779 |
| hsa-miR-548k | 8.63 ± 2.21 | 16.22 ± 2.66 | 1.99 | 0.1437 | 0.3779 |
| hsa-miR-182-5p | 7532.36 ± 1930.61 | 12360.82 ± 2141.27 | 1.72 | 0.1507 | 0.3864 |
| hsa-miR-132-3p | 40.22 ± 8.03 | 60.14 ± 7.31 | 1.55 | 0.1525 | 0.3864 |
| hsa-let-7b-5p | 7462.23 ± 1364.84 | 5020.52 ± 847.46 | 0.66 | 0.1533 | 0.3864 |
| hsa-miR-455-3p | 32.97 ± 5.82 | 49.87 ± 6.73 | 1.51 | 0.1535 | 0.3864 |
| hsa-miR-195-5p | 319.24 ± 71.73 | 177.32 ± 27.91 | 0.58 | 0.1546 | 0.3864 |
| hsa-miR-1180-3p | 9.8 ± 5.31 | 15.5 ± 4.64 | 2.96 | 0.1588 | 0.3931 |
| hsa-miR-3609 | 13.07 ± 4.44 | 7 ± 1.4 | 0.58 | 0.1673 | 0.4075 |
| hsa-miR-148b-5p | 10.42 ± 2.96 | 17.18 ± 1.49 | 1.81 | 0.1678 | 0.4075 |
| hsa-miR-30a-5p | 8490.51 ± 2177.6 | 5743.61 ± 2798.78 | 0.45 | 0.1711 | 0.4117 |
| hsa-miR-598-3p | 18.37 ± 4.58 | 30.46 ± 5.76 | 1.69 | 0.1759 | 0.4193 |
| hsa-miR-30c-5p | 944.34 ± 68.21 | 672.75 ± 55.07 | 0.69 | 0.1826 | 0.4198 |
| hsa-miR-34a-5p | 158.47 ± 19.44 | 111.59 ± 17.82 | 0.67 | 0.1828 | 0.4198 |
| hsa-miR-30e-3p | 401.51 ± 47 | 280.68 ± 7.28 | 0.70 | 0.1860 | 0.4198 |
| hsa-let-7a-5p | 43194.99 ± 13359.45 | 26742.95 ± 3023.48 | 0.69 | 0.1862 | 0.4198 |
| hsa-miR-374a-3p | 18.09 ± 7.92 | 37.01 ± 13.98 | 1.85 | 0.1872 | 0.4198 |
| hsa-miR-664a-3p | 40.75 ± 5.35 | 27.9 ± 5.57 | 0.63 | 0.1874 | 0.4198 |
| hsa-miR-203a-3p | 21958.17 ± 6178.13 | 10221.16 ± 3529.9 | 0.49 | 0.1877 | 0.4198 |
| hsa-miR-125b-1-3p | 41.62 ± 6.77 | 29.06 ± 6.55 | 0.62 | 0.1895 | 0.4202 |
| hsa-miR-145-5p | 510.96 ± 166.96 | 202.31 ± 27.33 | 0.48 | 0.1915 | 0.4209 |
| hsa-miR-181a-3p | 30.56 ± 5.26 | 47.04 ± 5.19 | 1.56 | 0.1946 | 0.4220 |
| hsa-miR-98-5p | 1064.56 ± 242.86 | 1606.87 ± 219.9 | 1.59 | 0.1953 | 0.4220 |
| hsa-miR-652-3p | 73.1 ± 9.31 | 51.21 ± 4.09 | 0.69 | 0.2030 | 0.4351 |
| hsa-miR-30d-5p | 5489.28 ± 706.88 | 3980.21 ± 229.58 | 0.72 | 0.2093 | 0.4448 |
| hsa-let-7e-5p | 482.43 ± 71.01 | 729.57 ± 118.94 | 1.45 | 0.2120 | 0.4468 |
| hsa-miR-330-5p | 10.86 ± 3.83 | 17.22 ± 3.56 | 1.88 | 0.2200 | 0.4599 |
| hsa-miR-200c-3p | 2121.6 ± 695.49 | 1254.51 ± 167.3 | 0.67 | 0.2223 | 0.4609 |
| hsa-miR-199b-5p | 299.94 ± 79.2 | 176.02 ± 55.68 | 0.48 | 0.2261 | 0.4650 |
| hsa-miR-221-3p | 2296.84 ± 354.31 | 1679.09 ± 236.03 | 0.71 | 0.2300 | 0.4693 |
| hsa-miR-5701 | 37.59 ± 6.5 | 29.58 ± 12.07 | 0.58 | 0.2361 | 0.4778 |
| hsa-miR-421 | 37.45 ± 5.81 | 64.03 ± 15.87 | 1.55 | 0.2384 | 0.4786 |
| hsa-miR-149-5p | 488.55 ± 98.86 | 324.57 ± 123.66 | 0.52 | 0.2406 | 0.4793 |
| hsa-miR-214-3p | 120.04 ± 32.6 | 70.85 ± 26.67 | 0.49 | 0.2460 | 0.4863 |
| hsa-miR-423-3p | 792.1 ± 271.08 | 464.2 ± 29.21 | 0.69 | 0.2529 | 0.4960 |
| hsa-miR-96-5p | 49.63 ± 13.38 | 69.7 ± 8.85 | 1.51 | 0.2559 | 0.4982 |
| hsa-miR-126-5p | 3162.53 ± 838.46 | 4198.53 ± 752 | 1.41 | 0.2581 | 0.4987 |
| hsa-miR-106b-5p | 46.61 ± 6.75 | 67.09 ± 12.92 | 1.38 | 0.2664 | 0.5108 |
| hsa-miR-148a-5p | 122.81 ± 16.63 | 180.82 ± 24.75 | 1.44 | 0.2685 | 0.5109 |
| hsa-miR-744-5p | 98.93 ± 39.4 | 54.24 ± 15.1 | 0.63 | 0.2727 | 0.5151 |
| hsa-miR-335-5p | 24.49 ± 4.09 | 18.47 ± 7.25 | 0.57 | 0.2846 | 0.5337 |
| hsa-miR-501-3p | 78.7 ± 13.13 | 58.01 ± 9.57 | 0.72 | 0.2883 | 0.5346 |
| hsa-miR-497-5p | 64.07 ± 16.22 | 34.01 ± 4.83 | 0.59 | 0.2893 | 0.5346 |
| hsa-miR-339-3p | 31.92 ± 8.14 | 42.75 ± 4.26 | 1.42 | 0.2972 | 0.5433 |
| hsa-miR-95-3p | 23.73 ± 7.45 | 14.78 ± 3.94 | 0.65 | 0.2983 | 0.5433 |
| hsa-miR-363-3p | 56.91 ± 15.69 | 43.29 ± 12.41 | 0.71 | 0.3024 | 0.5468 |
| hsa-miR-221-5p | 141.54 ± 40.98 | 90.29 ± 14.15 | 0.69 | 0.3197 | 0.5733 |
| hsa-miR-17-3p | 13.92 ± 2.85 | 20.48 ± 3.33 | 1.42 | 0.3224 | 0.5733 |
| hsa-miR-27b-5p | 66.32 ± 9.94 | 106.23 ± 31.41 | 1.39 | 0.3238 | 0.5733 |
| hsa-miR-128-3p | 78.39 ± 12.91 | 103.71 ± 8 | 1.35 | 0.3355 | 0.5868 |
| hsa-miR-203a-5p | 22.91 ± 8.71 | 13.31 ± 4.44 | 0.60 | 0.3407 | 0.5868 |
| hsa-miR-199a-5p | 550.12 ± 155.05 | 403.91 ± 162.53 | 0.56 | 0.3411 | 0.5868 |
| hsa-miR-23b-3p | 3790.95 ± 937.81 | 2803.4 ± 766.84 | 0.68 | 0.3422 | 0.5868 |
| hsa-let-7g-5p | 3757.92 ± 544.81 | 3087.08 ± 628.2 | 0.78 | 0.3438 | 0.5868 |
| hsa-miR-30d-3p | 16.42 ± 2.04 | 25.14 ± 5.15 | 1.40 | 0.3452 | 0.5868 |
| hsa-miR-365a-3p=hsa-miR-365b-3p | 92.7 ± 14.06 | 75.14 ± 18.96 | 0.74 | 0.3484 | 0.5884 |
| hsa-miR-155-5p | 364.84 ± 110.84 | 480.08 ± 98.25 | 1.41 | 0.3637 | 0.6102 |
| hsa-miR-140-3p | 567.74 ± 107.6 | 400.73 ± 29.53 | 0.74 | 0.3709 | 0.6143 |
| hsa-miR-205-5p | 44447.38 ± 12597.14 | 28214.16 ± 5999.26 | 0.70 | 0.3747 | 0.6143 |
| hsa-miR-222-3p | 1624 ± 553.22 | 1052.64 ± 181.75 | 0.72 | 0.3752 | 0.6143 |
| hsa-miR-374a-5p | 58.58 ± 16.42 | 71.48 ± 11.56 | 1.31 | 0.3786 | 0.6143 |
| hsa-miR-30a-3p | 176.1 ± 42.72 | 122.82 ± 39.44 | 0.61 | 0.3792 | 0.6143 |
| hsa-miR-101-3p | 477.32 ± 120.87 | 806.04 ± 238.92 | 1.49 | 0.3806 | 0.6143 |
| hsa-miR-339-5p | 25.92 ± 4.95 | 37.47 ± 7.43 | 1.39 | 0.3852 | 0.6153 |
| hsa-miR-181c-5p | 302.18 ± 49.09 | 492.5 ± 163.12 | 1.37 | 0.3861 | 0.6153 |
| hsa-miR-486-5p | 19837.89 ± 7404.77 | 13046.85 ± 3690.59 | 0.72 | 0.3953 | 0.6260 |
| hsa-miR-200b-5p | 27.37 ± 6.8 | 19.87 ± 2.52 | 0.76 | 0.4036 | 0.6353 |
| hsa-miR-151a-3p | 2796.98 ± 91.33 | 3643.48 ± 420.32 | 1.24 | 0.4188 | 0.6523 |
| hsa-miR-224-5p | 143.55 ± 32.93 | 210.7 ± 60.21 | 1.40 | 0.4202 | 0.6523 |
| hsa-miR-200a-5p | 42.66 ± 7.09 | 58.5 ± 11.09 | 1.32 | 0.4257 | 0.6523 |
| hsa-let-7i-5p | 3486.56 ± 396.96 | 4506.74 ± 521.12 | 1.26 | 0.4263 | 0.6523 |
| hsa-miR-92a-3p | 20455.97 ± 5486.94 | 15260.61 ± 2949.65 | 0.78 | 0.4272 | 0.6523 |
| hsa-miR-361-5p | 165.01 ± 26.44 | 131.25 ± 9.15 | 0.80 | 0.4326 | 0.6542 |
| hsa-miR-143-3p | 54213.04 ± 15592.31 | 67660.62 ± 9197.22 | 1.42 | 0.4337 | 0.6542 |
| hsa-miR-1296-5p | 20.76 ± 5.41 | 14.13 ± 2.48 | 0.73 | 0.4362 | 0.6542 |
| hsa-miR-532-3p | 15.99 ± 4.67 | 9.48 ± 1.26 | 0.69 | 0.4462 | 0.6643 |
| hsa-miR-144-5p | 22.8 ± 5.07 | 36.21 ± 14.48 | 1.37 | 0.4481 | 0.6643 |
| hsa-miR-25-3p | 1666.94 ± 74.2 | 2078.37 ± 124.28 | 1.21 | 0.4586 | 0.6745 |
| hsa-miR-199a-3p=hsa-miR-199b-3p | 2197.4 ± 593.51 | 1732.6 ± 578.99 | 0.66 | 0.4602 | 0.6745 |
| hsa-miR-29c-3p | 134.71 ± 42.38 | 203.64 ± 59.33 | 1.37 | 0.5036 | 0.7276 |
| hsa-miR-210-3p | 409.7 ± 93.73 | 287.4 ± 30.51 | 0.79 | 0.5061 | 0.7276 |
| hsa-miR-451a | 1477.66 ± 377.84 | 2081.76 ± 795.66 | 1.24 | 0.5078 | 0.7276 |
| hsa-miR-7974 | 11.95 ± 2.61 | 16.26 ± 3.45 | 1.30 | 0.5084 | 0.7276 |
| hsa-miR-1307-3p | 101.6 ± 36.04 | 113.13 ± 17.86 | 1.30 | 0.5141 | 0.7276 |
| hsa-miR-181c-3p | 22.26 ± 3.56 | 31.59 ± 7.3 | 1.30 | 0.5189 | 0.7276 |
| hsa-miR-126-3p | 959.52 ± 211.72 | 1133.4 ± 246.73 | 1.21 | 0.5226 | 0.7276 |
| hsa-miR-26b-5p | 2982.23 ± 133.98 | 2628.43 ± 338.33 | 0.83 | 0.5233 | 0.7276 |
| hsa-miR-27a-3p | 4930.36 ± 302.5 | 4368.17 ± 526.5 | 0.84 | 0.5244 | 0.7276 |
| hsa-miR-3615 | 33.2 ± 11.18 | 20.45 ± 2.78 | 0.74 | 0.5250 | 0.7276 |
| hsa-miR-22-3p | 13659.42 ± 3814.93 | 14974.87 ± 1056.43 | 1.20 | 0.5290 | 0.7288 |
| hsa-miR-183-5p | 2530.23 ± 741.46 | 3392.75 ± 957.75 | 1.36 | 0.5316 | 0.7288 |
| hsa-miR-30e-5p | 1687.36 ± 158.95 | 2000.86 ± 92.34 | 1.17 | 0.5394 | 0.7342 |
| hsa-miR-27b-3p | 47777.47 ± 8224.75 | 40067.39 ± 4785.72 | 0.83 | 0.5413 | 0.7342 |
| hsa-miR-145-3p | 75.91 ± 20.64 | 92.13 ± 17.15 | 1.29 | 0.5627 | 0.7591 |
| hsa-miR-7706 | 52.35 ± 17.94 | 63.31 ± 15.37 | 1.28 | 0.5724 | 0.7596 |
| hsa-miR-186-5p | 1826.47 ± 147.08 | 2197.89 ± 197.78 | 1.17 | 0.5725 | 0.7596 |
| hsa-miR-193a-3p | 20.35 ± 3.35 | 17.45 ± 3.19 | 0.84 | 0.5730 | 0.7596 |
| hsa-miR-197-3p | 240.57 ± 61.59 | 185.53 ± 11.84 | 0.84 | 0.5749 | 0.7596 |
| hsa-miR-345-5p | 101.29 ± 17.48 | 85.57 ± 7.01 | 0.86 | 0.5900 | 0.7755 |
| hsa-miR-629-5p | 23.08 ± 4.27 | 20.5 ± 3.9 | 0.85 | 0.6034 | 0.7816 |
| hsa-miR-582-3p | 19.06 ± 4.95 | 21.88 ± 3.79 | 1.17 | 0.6044 | 0.7816 |
| hsa-miR-589-5p | 41.49 ± 7.96 | 35.77 ± 4.48 | 0.86 | 0.6060 | 0.7816 |
| hsa-miR-409-3p | 53.37 ± 18.44 | 101.75 ± 53.5 | 1.57 | 0.6069 | 0.7816 |
| hsa-miR-191-5p | 21586.47 ± 3000.49 | 19010.72 ± 2229.81 | 0.87 | 0.6139 | 0.7866 |
| hsa-miR-181b-5p | 556.39 ± 70.78 | 507.82 ± 84.75 | 0.87 | 0.6188 | 0.7875 |
| hsa-miR-181a-5p | 11895.24 ± 994.63 | 11325.24 ± 2377.99 | 0.88 | 0.6208 | 0.7875 |
| hsa-miR-29a-3p | 772.01 ± 151.7 | 1019.89 ± 261.56 | 1.21 | 0.6288 | 0.7909 |
| hsa-miR-218-5p | 18.77 ± 6.07 | 28.77 ± 9 | 1.38 | 0.6296 | 0.7909 |
| hsa-miR-152-5p | 5.63 ± 1.83 | 6.25 ± 1.56 | 1.23 | 0.6358 | 0.7948 |
| hsa-miR-338-3p | 41.77 ± 19.76 | 28 ± 7.49 | 0.79 | 0.6581 | 0.8187 |
| hsa-miR-30b-5p | 339.89 ± 41.15 | 402.39 ± 54.64 | 1.14 | 0.6658 | 0.8228 |
| hsa-miR-484 | 259.3 ± 41.98 | 230.12 ± 22.61 | 0.89 | 0.6720 | 0.8228 |
| hsa-miR-584-5p | 34.45 ± 12.21 | 37.44 ± 11.18 | 1.21 | 0.6728 | 0.8228 |
| hsa-miR-152-3p | 251.91 ± 40.69 | 222.43 ± 30.72 | 0.87 | 0.6743 | 0.8228 |
| hsa-miR-16-5p | 4049.03 ± 438.91 | 4663.38 ± 476.54 | 1.13 | 0.6788 | 0.8243 |
| hsa-miR-361-3p | 53.19 ± 6.02 | 68.46 ± 18.74 | 1.14 | 0.6843 | 0.8271 |
| hsa-miR-24-3p | 2040.39 ± 112.02 | 1954.44 ± 296.72 | 0.90 | 0.6960 | 0.8371 |
| hsa-miR-148b-3p | 1720.81 ± 107.3 | 1630.93 ± 203.25 | 0.90 | 0.7065 | 0.8458 |
| hsa-miR-34c-5p | 44.49 ± 9.66 | 73.04 ± 31.58 | 1.20 | 0.7217 | 0.8599 |
| hsa-miR-660-5p | 85.92 ± 11.95 | 80.96 ± 13.71 | 0.91 | 0.7332 | 0.8696 |
| hsa-miR-425-5p | 143.76 ± 23.47 | 156.99 ± 12.15 | 1.11 | 0.7372 | 0.8703 |
| hsa-miR-532-5p | 245.03 ± 17.15 | 230.7 ± 20.27 | 0.92 | 0.7441 | 0.8714 |
| hsa-miR-331-3p | 53.64 ± 7.69 | 59.03 ± 6.68 | 1.10 | 0.7449 | 0.8714 |
| hsa-miR-374b-5p | 55.39 ± 7.46 | 51.37 ± 6.61 | 0.92 | 0.7602 | 0.8812 |
| hsa-miR-127-3p | 2109.74 ± 651.1 | 2140.61 ± 909.01 | 0.78 | 0.7632 | 0.8812 |
| hsa-miR-654-3p | 39.73 ± 14.44 | 74.48 ± 45.33 | 1.32 | 0.7637 | 0.8812 |
| hsa-miR-146a-5p | 4107.18 ± 2116.76 | 4406.6 ± 1828.73 | 1.17 | 0.7807 | 0.8871 |
| hsa-miR-362-5p | 28.84 ± 2.77 | 32.2 ± 3.17 | 1.08 | 0.7827 | 0.8871 |
| hsa-miR-193a-5p | 41.5 ± 8.52 | 39.47 ± 8.7 | 0.90 | 0.7836 | 0.8871 |
| hsa-miR-1307-5p | 246.88 ± 34.93 | 265.4 ± 19.25 | 1.08 | 0.7901 | 0.8871 |
| hsa-let-7d-3p | 113.25 ± 14.13 | 105.67 ± 7.63 | 0.93 | 0.7949 | 0.8871 |
| hsa-miR-193b-3p | 353.41 ± 86.58 | 334.31 ± 68.68 | 0.93 | 0.7985 | 0.8871 |
| hsa-miR-93-5p | 643.59 ± 70.5 | 610.97 ± 65.42 | 0.93 | 0.8023 | 0.8871 |
| hsa-miR-26b-3p | 14.85 ± 5.86 | 13.78 ± 2.12 | 1.12 | 0.8036 | 0.8871 |
| hsa-miR-150-5p | 751.29 ± 254.71 | 752.18 ± 244.52 | 0.90 | 0.8040 | 0.8871 |
| hsa-miR-320a | 900.26 ± 195.18 | 793.85 ± 58.66 | 0.93 | 0.8045 | 0.8871 |
| hsa-miR-106b-3p | 142.54 ± 14.92 | 134.68 ± 8.1 | 0.94 | 0.8071 | 0.8871 |
| hsa-miR-769-5p | 229.8 ± 23.78 | 221.67 ± 29.19 | 0.93 | 0.8177 | 0.8930 |
| hsa-miR-141-3p | 4921.35 ± 591 | 5866.56 ± 2361.75 | 0.92 | 0.8194 | 0.8930 |
| hsa-miR-181d-5p | 44.32 ± 11.46 | 53.53 ± 16.81 | 1.11 | 0.8291 | 0.8958 |
| hsa-miR-23a-3p | 1674.5 ± 225.86 | 1586.08 ± 141.98 | 0.94 | 0.8299 | 0.8958 |
| hsa-miR-103a-3p | 1621.07 ± 145.61 | 1558.68 ± 122.76 | 0.94 | 0.8326 | 0.8958 |
| hsa-miR-28-3p | 1936.17 ± 248.22 | 2077.27 ± 262.82 | 1.05 | 0.8479 | 0.9085 |
| hsa-miR-342-3p | 576.72 ± 130.27 | 538.84 ± 101.52 | 0.94 | 0.8531 | 0.9102 |
| hsa-let-7d-5p | 454.3 ± 67.48 | 436.38 ± 33.55 | 0.96 | 0.8786 | 0.9335 |
| hsa-miR-200b-3p | 1517.34 ± 348.31 | 1578.01 ± 304.04 | 1.04 | 0.8924 | 0.9406 |
| hsa-miR-151a-5p | 1138.39 ± 63.87 | 1171.61 ± 166.88 | 0.96 | 0.8937 | 0.9406 |
| hsa-miR-151b | 1132.83 ± 63.7 | 1167.54 ± 166.95 | 0.97 | 0.8964 | 0.9406 |
| hsa-miR-99b-5p | 4909.19 ± 613.96 | 5224.03 ± 775.49 | 1.03 | 0.9030 | 0.9437 |
| hsa-miR-107 | 283.16 ± 6.61 | 286.33 ± 20.1 | 0.98 | 0.9302 | 0.9681 |
| hsa-let-7f-5p | 9448.69 ± 1968.02 | 9398.78 ± 1362.13 | 1.02 | 0.9462 | 0.9808 |
| hsa-miR-671-3p | 31.54 ± 13.36 | 26.07 ± 2.54 | 1.02 | 0.9512 | 0.9820 |
| hsa-miR-941 | 743.65 ± 246.68 | 648.8 ± 117.52 | 0.99 | 0.9678 | 0.9856 |
| hsa-miR-335-3p | 113.11 ± 9.34 | 121.04 ± 21.95 | 0.99 | 0.9680 | 0.9856 |
| hsa-miR-423-5p | 338 ± 89 | 311.53 ± 35.02 | 0.99 | 0.9683 | 0.9856 |
| hsa-miR-324-5p | 21.83 ± 1.98 | 22.61 ± 2.08 | 1.01 | 0.9702 | 0.9856 |
| hsa-let-7b-3p | 98.34 ± 18.26 | 100.76 ± 17.06 | 1.01 | 0.9799 | 0.9870 |
| hsa-miR-15b-5p | 203.86 ± 46.82 | 195.12 ± 20.85 | 1.01 | 0.9799 | 0.9870 |
| hsa-miR-28-5p | 190.9 ± 14.42 | 200.55 ± 24.08 | 1.01 | 0.9831 | 0.9870 |
| hsa-miR-411-5p | 51.24 ± 13.86 | 64.88 ± 22.68 | 1.01 | 0.9935 | 0.9935 |

Supplemental Table 2 – MicroRNAs expressed in the serum of patients diagnosed with HNSCC.

| miRNA | CPM |
| --- | --- |
| hsa-miR-486-5p | 1330484.82 ± 221358.01 |
| hsa-miR-22-3p | 265366.76 ± 34181.03 |
| hsa-miR-92a-3p | 208058.62 ± 27039.27 |
| hsa-miR-16-5p | 170507.59 ± 37999.33 |
| hsa-miR-451a | 120792.41 ± 38100.08 |
| hsa-miR-423-5p | 70826.46 ± 6539.78 |
| hsa-miR-27b-3p | 41121.98 ± 12104.9 |
| hsa-miR-142-5p | 38076.31 ± 6305.79 |
| hsa-miR-26a-5p | 35034.09 ± 8275.06 |
| hsa-miR-10b-5p | 33464.52 ± 3329.98 |
| hsa-miR-192-5p | 32709.39 ± 12257.74 |
| hsa-miR-25-3p | 32581.79 ± 6003.76 |
| hsa-miR-126-5p | 28135.61 ± 1493.93 |
| hsa-miR-191-5p | 27685.9 ± 4711.44 |
| hsa-miR-30d-5p | 25699.68 ± 2144.09 |
| hsa-miR-30e-5p | 22238.44 ± 1831.68 |
| hsa-miR-148a-3p | 22047.67 ± 6552.44 |
| hsa-miR-181a-5p | 18260.07 ± 1794 |
| hsa-miR-10a-5p | 17496 ± 1805.61 |
| hsa-miR-21-5p | 17457.05 ± 845.78 |
| hsa-miR-103a-3p | 14369.24 ± 2708.39 |
| hsa-miR-107 | 12739.52 ± 2908.79 |
| hsa-miR-15a-5p | 11607.99 ± 3249.71 |
| hsa-miR-223-3p | 11337.54 ± 1878.43 |
| hsa-let-7f-5p | 11049.64 ± 1065.66 |
| hsa-miR-186-5p | 9094.14 ± 1139.1 |
| hsa-miR-151a-3p | 8981.37 ± 556.79 |
| hsa-miR-143-3p | 8742.84 ± 1932.42 |
| hsa-miR-423-3p | 7992.78 ± 513.18 |
| hsa-let-7a-5p | 7096.2 ± 558.84 |
| hsa-miR-30a-5p | 6844.86 ± 1551.5 |
| hsa-miR-320a | 6816.78 ± 695.04 |
| hsa-miR-101-3p | 6711.59 ± 1523.37 |
| hsa-let-7i-5p | 6120.38 ± 377.96 |
| hsa-miR-150-5p | 6070.31 ± 1978.45 |
| hsa-miR-378a-3p | 5936.03 ± 1485.16 |
| hsa-miR-484 | 5529.12 ± 987.9 |
| hsa-miR-425-5p | 5458.22 ± 424.32 |
| hsa-miR-27a-3p | 5211.82 ± 493.48 |
| hsa-miR-1246 | 4861.62 ± 1224.89 |
| hsa-miR-363-3p | 4431.79 ± 1132.71 |
| hsa-miR-28-3p | 4341.15 ± 425.54 |
| hsa-miR-146a-5p | 4330.36 ± 909.03 |
| hsa-miR-151a-5p | 4282.33 ± 1063.3 |
| hsa-miR-151b | 4274.11 ± 1059.34 |
| hsa-miR-140-3p | 4170.91 ± 641.69 |
| hsa-miR-93-5p | 3997.26 ± 559.36 |
| hsa-miR-130a-3p | 3759 ± 912.64 |
| hsa-miR-182-5p | 3734.51 ± 862.92 |
| hsa-miR-126-3p | 3555.72 ± 271.16 |
| hsa-miR-375 | 3171.1 ± 1400.83 |
| hsa-miR-199a-3p=hsa-miR-199b-3p | 3052.36 ± 204.71 |
| hsa-miR-221-3p | 3031.24 ± 87.4 |
| hsa-miR-23a-3p | 2876.02 ± 158.33 |
| hsa-let-7b-5p | 2835.42 ± 429.28 |
| hsa-miR-16-2-3p | 2774.4 ± 783.77 |
| hsa-miR-320b | 2613.82 ± 585.86 |
| hsa-miR-26b-5p | 2510.97 ± 292.2 |
| hsa-miR-4532 | 2488.65 ± 913.75 |
| hsa-let-7d-3p | 2465.82 ± 425.68 |
| hsa-miR-92b-3p | 2434.11 ± 354.25 |
| hsa-miR-122-5p | 2379.42 ± 990.29 |
| hsa-miR-30c-5p | 2316.34 ± 478.28 |
| hsa-miR-1307-5p | 2294.61 ± 285.74 |
| hsa-miR-584-5p | 2248.99 ± 253.78 |
| hsa-miR-29a-3p | 2132.44 ± 380.75 |
| hsa-miR-127-3p | 1897.8 ± 824.17 |
| hsa-miR-106b-3p | 1876.1 ± 318.22 |
| hsa-let-7g-5p | 1866.61 ± 55.94 |
| hsa-miR-486-3p | 1750.16 ± 389.24 |
| hsa-miR-744-5p | 1645.06 ± 241.93 |
| hsa-miR-21-3p | 1589.63 ± 51.02 |
| hsa-miR-128-3p | 1572.96 ± 262.72 |
| hsa-let-7d-5p | 1571.99 ± 321.21 |
| hsa-miR-222-3p | 1460.45 ± 159.82 |
| hsa-miR-532-5p | 1452.29 ± 226.55 |
| hsa-miR-148b-3p | 1347.18 ± 326.93 |
| hsa-miR-125a-5p | 1335.16 ± 171.93 |
| hsa-miR-345-5p | 1325.54 ± 248.07 |
| hsa-miR-19b-3p | 1259.81 ± 272.98 |
| hsa-miR-144-3p | 1191.97 ± 206.91 |
| hsa-miR-130b-3p | 1175.18 ± 184.27 |
| hsa-miR-23b-3p | 1150.13 ± 98.94 |
| hsa-miR-100-5p | 1122.82 ± 304.58 |
| hsa-miR-99b-5p | 1111.24 ± 100.09 |
| hsa-miR-652-3p | 1093.99 ± 278.66 |
| hsa-miR-3615 | 1085.91 ± 139.2 |
| hsa-miR-146b-5p | 902.41 ± 130.94 |
| hsa-miR-210-3p | 843.94 ± 130.88 |
| hsa-miR-941 | 801.29 ± 117.77 |
| hsa-miR-29c-3p | 782.4 ± 134.56 |
| hsa-miR-409-3p | 777.62 ± 261.04 |
| hsa-miR-24-3p | 767.4 ± 180.41 |
| hsa-miR-30b-5p | 740.44 ± 140.97 |
| hsa-miR-106b-5p | 722.4 ± 143.79 |
| hsa-miR-181b-5p | 701.65 ± 81.28 |
| hsa-miR-654-3p | 679.26 ± 224.53 |
| hsa-miR-144-5p | 664.4 ± 95.68 |
| hsa-miR-17-5p | 663.39 ± 43.66 |
| hsa-miR-4732-3p | 624.59 ± 122.48 |
| hsa-miR-342-3p | 608.65 ± 94.97 |
| hsa-miR-15b-5p | 590.72 ± 65.43 |
| hsa-miR-340-5p | 558.6 ± 49.51 |
| hsa-miR-197-3p | 552.48 ± 86.64 |
| hsa-miR-421 | 547.98 ± 53.18 |
| hsa-miR-133a-3p | 532.84 ± 404.99 |
| hsa-miR-660-5p | 528.29 ± 68.76 |
| hsa-miR-125b-2-3p | 482.29 ± 96.32 |
| hsa-miR-99a-5p | 460.9 ± 135.87 |
| hsa-miR-339-3p | 437.4 ± 80.71 |
| hsa-miR-361-5p | 435.01 ± 45.61 |
| hsa-miR-335-5p | 434.52 ± 29.2 |
| hsa-miR-574-3p | 426.56 ± 89.19 |
| hsa-miR-1307-3p | 425.43 ± 24.09 |
| hsa-miR-142-3p | 410.69 ± 41.15 |
| hsa-miR-769-5p | 382.41 ± 46.71 |
| hsa-miR-424-3p | 376.07 ± 44.27 |
| hsa-miR-181c-5p | 363.62 ± 43.64 |
| hsa-miR-500a-3p | 353.79 ± 36.84 |
| hsa-miR-20a-5p | 352.03 ± 66.84 |
| hsa-miR-183-5p | 342.12 ± 98.64 |
| hsa-let-7e-5p | 332.25 ± 58.68 |
| hsa-miR-589-5p | 326.55 ± 47.38 |
| hsa-miR-410-3p | 325.89 ± 79.26 |
| hsa-miR-194-5p | 322.33 ± 100.83 |
| hsa-miR-32-5p | 317.32 ± 84.4 |
| hsa-miR-98-5p | 316.85 ± 54.11 |
| hsa-miR-30e-3p | 314.9 ± 42.83 |
| hsa-miR-19a-3p | 296.55 ± 63.03 |
| hsa-miR-28-5p | 279.85 ± 45.01 |
| hsa-miR-125b-5p | 274.03 ± 81.28 |
| hsa-miR-148a-5p | 261.5 ± 67.26 |
| hsa-miR-199a-5p | 258.11 ± 64.09 |
| hsa-miR-328-3p | 256.38 ± 37.59 |
| hsa-let-7c-5p | 239.67 ± 46.83 |
| hsa-miR-155-5p | 235.8 ± 70.05 |
| hsa-miR-301a-3p | 234.56 ± 22.8 |
| hsa-miR-4433b-5p | 232.77 ± 103.59 |
| hsa-miR-877-5p | 231.39 ± 21.11 |
| hsa-let-7b-3p | 226.98 ± 38.49 |
| hsa-miR-885-5p | 218.35 ± 68.91 |
| hsa-miR-181a-2-3p | 204.13 ± 15.13 |
| hsa-miR-26b-3p | 203.36 ± 12.48 |
| hsa-miR-205-5p | 200.62 ± 92.74 |
| hsa-miR-4508 | 199.09 ± 59.35 |
| hsa-miR-215-5p | 196.91 ± 93.23 |
| hsa-miR-15b-3p | 190.69 ± 54.79 |
| hsa-miR-203a-3p | 176.86 ± 46.31 |
| hsa-miR-361-3p | 174.61 ± 24.3 |
| hsa-miR-223-5p | 173.13 ± 27 |
| hsa-miR-501-3p | 171.17 ± 10.62 |
| hsa-miR-6087 | 169.48 ± 30.44 |
| hsa-miR-342-5p | 168.31 ± 30.09 |
| hsa-miR-141-3p | 168.17 ± 47.5 |
| hsa-miR-378c | 166.04 ± 42.41 |
| hsa-miR-411-5p | 154.87 ± 71.76 |
| hsa-miR-942-5p | 154.38 ± 22.95 |
| hsa-miR-339-5p | 153.58 ± 27.79 |
| hsa-miR-502-3p | 149.42 ± 18.27 |
| hsa-miR-450b-5p | 145.1 ± 36.83 |
| hsa-miR-431-5p | 141.9 ± 41.92 |
| hsa-miR-326 | 138.41 ± 17.76 |
| hsa-miR-425-3p | 136.78 ± 32.43 |
| hsa-miR-335-3p | 135.26 ± 36.44 |
| hsa-miR-324-3p | 128.39 ± 24.08 |
| hsa-miR-136-3p | 124.02 ± 39.58 |
| hsa-miR-30a-3p | 123.96 ± 18.25 |
| hsa-miR-671-3p | 123.32 ± 27.58 |
| hsa-miR-181a-3p | 121.07 ± 29.68 |
| hsa-miR-7706 | 119.75 ± 17.12 |
| hsa-miR-1468-5p | 119.47 ± 27.52 |
| hsa-miR-550a-3p | 117.78 ± 19.27 |
| hsa-miR-130b-5p | 116.28 ± 23 |
| hsa-miR-3158-3p | 113.92 ± 15.08 |
| hsa-miR-432-5p | 113.21 ± 27.97 |
| hsa-miR-6852-5p | 112.07 ± 17.99 |
| hsa-miR-152-3p | 106.79 ± 16.85 |
| hsa-miR-132-3p | 104.72 ± 18.89 |
| hsa-miR-381-3p | 103.17 ± 30.82 |
| hsa-miR-505-3p | 103.01 ± 19.47 |
| hsa-miR-93-3p | 102.74 ± 11.27 |
| hsa-miR-576-5p | 98.92 ± 11.14 |
| hsa-miR-33b-5p | 97.83 ± 30.61 |
| hsa-miR-181c-3p | 97.18 ± 19.36 |
| hsa-miR-193b-5p | 95.14 ± 33.8 |
| hsa-miR-4492 | 90.86 ± 11.28 |
| hsa-miR-374b-5p | 90.86 ± 9.19 |
| hsa-let-7i-3p | 90.54 ± 24.5 |
| hsa-miR-4446-3p | 89.34 ± 19.42 |
| hsa-miR-17-3p | 84.8 ± 16.29 |
| hsa-miR-323b-3p | 82.78 ± 27.76 |
| hsa-miR-18a-3p | 81.95 ± 21.28 |
| hsa-miR-191-3p | 79.51 ± 17.52 |
| hsa-miR-497-5p | 76.66 ± 11.72 |
| hsa-miR-889-3p | 75.43 ± 21.28 |
| hsa-miR-874-3p | 73.57 ± 11.21 |
| hsa-let-7a-3p | 73.4 ± 18.04 |
| hsa-miR-3605-3p | 70.8 ± 18.78 |
| hsa-miR-224-5p | 70.37 ± 17.18 |
| hsa-miR-146b-3p | 70.05 ± 9.95 |
| hsa-miR-338-3p | 69.92 ± 6.06 |
| hsa-miR-18a-5p | 67.84 ± 9.88 |
| hsa-miR-193b-3p | 67.49 ± 28.16 |
| hsa-miR-454-5p | 67.39 ± 8.49 |
| hsa-miR-582-3p | 66.12 ± 24.51 |
| hsa-miR-532-3p | 65.85 ± 12 |
| hsa-miR-20b-5p | 65.18 ± 17.55 |
| hsa-miR-365a-3p=hsa-miR-365b-3p | 62.57 ± 22.53 |
| hsa-miR-134-5p | 62.36 ± 22.09 |
| hsa-miR-185-3p | 62.29 ± 6.93 |
| hsa-miR-1180-3p | 61.72 ± 16.92 |
| hsa-miR-628-3p | 59.81 ± 4.49 |
| hsa-miR-5010-5p | 59.61 ± 12.21 |
| hsa-miR-550a-3-5p | 59.48 ± 3.93 |
| hsa-miR-550a-5p | 59.48 ± 3.93 |
| hsa-miR-96-5p | 58.52 ± 23.82 |
| hsa-miR-145-3p | 58.52 ± 8.38 |
| hsa-miR-374a-5p | 58.48 ± 7.79 |
| hsa-miR-330-3p | 58.45 ± 13.77 |
| hsa-miR-106a-5p | 57.1 ± 16.71 |
| hsa-miR-625-3p | 57.08 ± 5.78 |
| hsa-miR-199b-5p | 56.64 ± 7.09 |
| hsa-miR-371b-5p | 56.6 ± 14.52 |
| hsa-miR-625-5p | 55.83 ± 11.4 |
| hsa-miR-4516 | 55.42 ± 12.07 |
| hsa-miR-22-5p | 54.64 ± 10 |
| hsa-miR-320c | 51.97 ± 13.13 |
| hsa-miR-204-5p | 51.85 ± 11.58 |
| hsa-miR-424-5p | 51.61 ± 12.22 |
| hsa-miR-181d-5p | 51.09 ± 7.77 |
| hsa-miR-139-5p | 49.49 ± 14.55 |
| hsa-miR-483-5p | 48.78 ± 17.01 |
| hsa-miR-6842-3p | 47.6 ± 6.47 |
| hsa-miR-454-3p | 47.41 ± 6.09 |
| hsa-miR-150-3p | 46.24 ± 12.94 |
| hsa-miR-30d-3p | 45.82 ± 6.9 |
| hsa-miR-627-5p | 44.84 ± 9.05 |
| hsa-miR-629-5p | 43.47 ± 11.56 |
| hsa-miR-4732-5p | 43.32 ± 10.76 |
| hsa-miR-340-3p | 42.63 ± 10.38 |
| hsa-miR-320d | 41.48 ± 6.68 |
| hsa-miR-301b-3p | 41.19 ± 4.73 |
| hsa-miR-25-5p | 40.68 ± 5.8 |
| hsa-miR-145-5p | 40.37 ± 7.33 |
| hsa-miR-4662a-5p | 39.56 ± 2.63 |
| hsa-miR-148b-5p | 39.43 ± 7.23 |
| hsa-miR-324-5p | 37.58 ± 7.04 |
| hsa-miR-483-3p | 35.63 ± 8.85 |
| hsa-miR-3909 | 35.61 ± 10.83 |
| hsa-miR-5189-5p | 35.1 ± 3.58 |
| hsa-miR-598-3p | 33.85 ± 3.94 |
| hsa-miR-188-5p | 32.49 ± 5 |
| hsa-miR-6511a-3p | 31.99 ± 7.08 |
| hsa-miR-323a-3p | 31.49 ± 6.16 |
| hsa-let-7f-2-3p | 31.29 ± 8.01 |
| hsa-miR-221-5p | 30.63 ± 10.08 |
| hsa-miR-450a-5p | 30.43 ± 6.53 |
| hsa-miR-7704 | 29.25 ± 5.11 |
| hsa-miR-101-5p | 29.25 ± 6.99 |
| hsa-miR-185-5p | 29.21 ± 7.55 |
| hsa-miR-30c-1-3p | 28.07 ± 3.54 |
| hsa-miR-548o-3p | 27.87 ± 9.12 |
| hsa-let-7f-1-3p | 27.78 ± 3.71 |
| hsa-miR-582-5p | 27.61 ± 4.65 |
| hsa-miR-548av-5p | 26.64 ± 4.42 |
| hsa-miR-548k | 26.64 ± 4.42 |
| hsa-miR-140-5p | 26.26 ± 2.82 |
| hsa-miR-2110 | 25.59 ± 4.05 |
| hsa-miR-331-5p | 25.52 ± 3.54 |
| hsa-miR-98-3p | 25.5 ± 6.33 |
| hsa-miR-195-5p | 25.49 ± 3.87 |
| hsa-miR-548e-3p | 25.24 ± 4.53 |
| hsa-miR-29b-3p | 25.11 ± 4.64 |
| hsa-miR-548e-5p | 25.08 ± 2.57 |
| hsa-miR-7-1-3p | 24.62 ± 6.67 |
| hsa-miR-654-5p | 24.22 ± 9.98 |
| hsa-miR-511-5p | 23.98 ± 4.82 |
| hsa-miR-3688-3p | 23.88 ± 7.95 |
| hsa-miR-1301-3p | 23.22 ± 3.15 |
| hsa-miR-616-5p | 22.94 ± 5.45 |
| hsa-miR-196b-5p | 22.33 ± 4 |
| hsa-miR-374a-3p | 21.92 ± 6.25 |
| hsa-miR-200b-3p | 21.87 ± 11.45 |
| hsa-miR-4488 | 20.85 ± 6.62 |
| hsa-miR-4326 | 20.53 ± 2.85 |
| hsa-miR-10a-3p | 20.16 ± 2.51 |
| hsa-miR-330-5p | 19.97 ± 5.46 |
| hsa-miR-369-3p | 19.13 ± 2.2 |
| hsa-miR-2355-5p | 19.03 ± 4.25 |
| hsa-miR-3176 | 18.5 ± 3.18 |
| hsa-miR-664a-3p | 17.38 ± 2.36 |
| hsa-miR-452-5p | 17.31 ± 6.14 |
| hsa-miR-1976 | 15.88 ± 1.32 |
| hsa-miR-3173-5p | 15.52 ± 2.04 |
| hsa-miR-27b-5p | 15.36 ± 4.05 |
| hsa-miR-331-3p | 14.84 ± 1.81 |
| hsa-miR-378a-5p | 14.51 ± 3.29 |
| hsa-miR-18b-5p | 14.46 ± 3.14 |
| hsa-miR-1260b | 14.19 ± 3 |
| hsa-miR-873-3p | 13.38 ± 3.88 |
| hsa-miR-195-3p | 13.11 ± 1.19 |
| hsa-miR-664a-5p | 13.07 ± 1.52 |
| hsa-miR-6741-3p | 12.71 ± 3.98 |
| hsa-miR-744-3p | 11.02 ± 1.47 |
| hsa-miR-10b-3p | 11.02 ± 1.64 |
| hsa-miR-589-3p | 10.79 ± 1.49 |
| hsa-miR-3120-3p | 10.69 ± 1.47 |
| hsa-miR-6511b-3p | 9.66 ± 1.23 |
| hsa-miR-30c-2-3p | 9.19 ± 1.54 |
| hsa-miR-200a-3p | 70.96 ± 51.81 |
| hsa-miR-429 | 69.74 ± 55.17 |
| hsa-miR-487b-3p | 46.49 ± 24.95 |
| hsa-miR-338-5p | 42.12 ± 15 |
| hsa-miR-485-5p | 38.5 ± 21.85 |
| hsa-miR-370-3p | 33.07 ± 11.8 |
| hsa-miR-3200-3p | 27.86 ± 6.58 |
| hsa-miR-24-2-5p | 27.54 ± 7.29 |
| hsa-miR-369-5p | 27.46 ± 11.92 |
| hsa-miR-543 | 27.19 ± 13.1 |
| hsa-miR-3605-5p | 24.13 ± 6.86 |
| hsa-miR-3614-5p | 23.93 ± 10.29 |
| hsa-miR-493-3p | 23.88 ± 8.87 |
| hsa-miR-758-3p | 22.63 ± 11.94 |
| hsa-miR-766-3p | 22.06 ± 8.93 |
| hsa-miR-125b-1-3p | 19.85 ± 6.05 |
| hsa-miR-485-3p | 19.46 ± 9.51 |
| hsa-miR-128-1-5p | 18.69 ± 6.65 |
| hsa-miR-7976 | 17.23 ± 5.42 |
| hsa-miR-5001-3p | 17.22 ± 5.31 |
| hsa-miR-1287-5p | 16 ± 5.13 |
| hsa-miR-33a-5p | 13.97 ± 5.83 |
| hsa-miR-377-5p | 13.55 ± 4.41 |
| hsa-miR-193a-3p | 12.68 ± 3.51 |
| hsa-miR-494-3p | 12.61 ± 4.5 |
| hsa-miR-636 | 12.51 ± 3.52 |
| hsa-miR-3157-3p | 11.7 ± 3.05 |
| hsa-miR-4746-5p | 11.56 ± 2.94 |
| hsa-miR-937-3p | 11.16 ± 3.14 |
| hsa-miR-3200-5p | 11.15 ± 3.71 |
| hsa-miR-873-5p | 10.69 ± 3.4 |
| hsa-miR-656-3p | 10.43 ± 3.65 |
| hsa-miR-6513-3p | 10.1 ± 2.33 |
| hsa-miR-5588-5p | 9.95 ± 2.97 |
| hsa-miR-95-3p | 9.58 ± 4.33 |
| hsa-miR-1294 | 9.44 ± 3.93 |
| hsa-miR-1538 | 9.39 ± 1.5 |
| hsa-miR-1185-1-3p | 9.13 ± 3.44 |
| hsa-miR-3656 | 8.65 ± 3.01 |
| hsa-miR-18b-3p | 8.58 ± 1.87 |
| hsa-miR-1304-5p | 8.4 ± 1.73 |
| hsa-miR-200c-3p | 7.77 ± 1.61 |
| hsa-miR-652-5p | 7.31 ± 1.37 |
| hsa-miR-627-3p | 6.04 ± 1.23 |
| hsa-miR-1270 | 6 ± 1.12 |
| hsa-miR-6777-3p | 5.55 ± 1.55 |
| hsa-miR-433-3p | 20.99 ± 9.3 |
| hsa-miR-499a-5p | 17.51 ± 8.09 |
| hsa-miR-214-3p | 16.06 ± 6.6 |
| hsa-miR-3143 | 14.37 ± 4.72 |
| hsa-miR-1296-5p | 11.16 ± 4.02 |
| hsa-let-7g-3p | 9.92 ± 3.57 |
| hsa-miR-651-5p | 9.15 ± 4.88 |
| hsa-miR-337-3p | 9.12 ± 3.97 |
| hsa-miR-1254 | 8.13 ± 2.23 |
| hsa-miR-212-3p | 8.02 ± 2.56 |
| hsa-miR-503-5p | 7.78 ± 2.46 |
| hsa-miR-4511 | 7.61 ± 2.54 |
| hsa-miR-2115-3p | 7.41 ± 3.11 |
| hsa-miR-4645-3p | 7.14 ± 1.37 |
| hsa-miR-628-5p | 7 ± 2.42 |
| hsa-miR-4685-3p | 6.99 ± 1.54 |
| hsa-miR-5683 | 6.99 ± 1.62 |
| hsa-miR-136-5p | 6.94 ± 1.9 |
| hsa-miR-490-3p | 6.67 ± 2.01 |
| hsa-miR-3177-3p | 6.46 ± 2.08 |
| hsa-miR-760 | 6.28 ± 1.09 |
| hsa-miR-3613-3p | 6.2 ± 0.92 |
| hsa-miR-1306-3p | 5.97 ± 1.81 |
| hsa-miR-190a-5p | 5.49 ± 1.62 |
| hsa-miR-496 | 5.46 ± 2.29 |
| hsa-miR-2355-3p | 4.91 ± 2.09 |

Supplemental Table 3 – MicroRNAs differentially expressed between tumor and healthy adjacent tissue and also found in the serum samples of patients diagnosed with HNSCC.

|  |  | Tissue | |
| --- | --- | --- | --- |
| microRNA | Serum | Healthy | Tumor |
| hsa-miR-3656 | 8.65 ± 3.01 | 622.23 ± 139.7 | 202.24 ± 92.26 |
| hsa-miR-4508 | 199.09 ± 59.35 | 513.24 ± 82.05 | 165.24 ± 87.58 |
| hsa-miR-92b-3p | 2434.11 ± 354.25 | 497.22 ± 94.34 | 1465.13 ± 210.46 |
| hsa-miR-125b-5p | 274.03 ± 81.28 | 2016.76 ± 257.48 | 619.77 ± 211.97 |
| hsa-miR-18a-5p | 67.84 ± 9.88 | 24.29 ± 5.7 | 123.25 ± 36.61 |
| hsa-let-7a-3p | 73.4 ± 18.04 | 23.56 ± 16.17 | 52.02 ± 10.75 |
| hsa-miR-4488 | 20.85 ± 6.62 | 489.12 ± 102.88 | 187.6 ± 85.58 |
| hsa-miR-99a-5p | 460.9 ± 135.87 | 1185.4 ± 41.23 | 395.01 ± 93.85 |
| hsa-miR-424-5p | 51.61 ± 12.22 | 2.14 ± 1.86 | 39.75 ± 7.37 |
| hsa-miR-375 | 3171.1 ± 1400.83 | 8515.05 ± 3731.04 | 1026.33 ± 475.78 |
| hsa-miR-223-3p | 11337.54 ± 1878.43 | 63.71 ± 13.03 | 442.93 ± 139.65 |
| hsa-miR-142-5p | 38076.31 ± 6305.79 | 816.58 ± 219.92 | 2412.08 ± 695.48 |
| hsa-miR-139-5p | 49.49 ± 14.55 | 77.3 ± 10.43 | 21.21 ± 4.97 |
| hsa-miR-877-5p | 231.39 ± 21.11 | 1.27 ± 1.09 | 18.72 ± 5.12 |
| hsa-miR-19a-3p | 296.55 ± 63.03 | 6.74 ± 3.3 | 33.87 ± 10.76 |
| hsa-miR-7704 | 29.25 ± 5.11 | 660.64 ± 175.7 | 219.49 ± 129.52 |
| hsa-miR-32-5p | 317.32 ± 84.4 | 5.49 ± 2.43 | 24.26 ± 6.04 |
| hsa-miR-196b-5p | 22.33 ± 4 | 2.02 ± 1.75 | 30.15 ± 10.01 |
| hsa-miR-4532 | 2488.65 ± 913.75 | 5351.31 ± 955.34 | 1714.59 ± 702 |
| hsa-let-7c-5p | 239.67 ± 46.83 | 4812.94 ± 1102.21 | 1129.95 ± 257.38 |
| hsa-miR-4516 | 55.42 ± 12.07 | 1635 ± 411.42 | 504.11 ± 268.88 |
| hsa-miR-204-5p | 51.85 ± 11.58 | 344.22 ± 91.11 | 17.55 ± 4 |
| hsa-miR-4492 | 90.86 ± 11.28 | 2079.89 ± 449.49 | 623.76 ± 345.65 |
| hsa-miR-125b-2-3p | 482.29 ± 96.32 | 632.01 ± 120.54 | 169.59 ± 28.9 |
| hsa-miR-6087 | 169.48 ± 30.44 | 863.11 ± 228.33 | 197.8 ± 98.4 |
| hsa-miR-21-5p | 17457.05 ± 845.78 | 11577 ± 2360.39 | 40506.05 ± 5524.39 |
| hsa-miR-20a-5p | 352.03 ± 66.84 | 87.07 ± 12.5 | 214.73 ± 26.33 |
| hsa-miR-21-3p | 1589.63 ± 51.02 | 1366.33 ± 343.19 | 3663.11 ± 527.37 |
| hsa-miR-142-3p | 410.69 ± 41.15 | 12.2 ± 6.43 | 94.3 ± 26.39 |
| hsa-miR-301a-3p | 234.56 ± 22.8 | 9.17 ± 2.54 | 46.43 ± 12.17 |

Supplemental Table 4 – Pathways of target genes from the 48 miRNAs differentially expressed between tumor and healthy tissue of HNSCC patients.

| KEGG pathway | P value | Genes | miRNAs |
| --- | --- | --- | --- |
| Hippo signaling pathway | 8.98E-09 | 102 | 33 |
| Signaling pathways regulating pluripotency of stem cells | 2.18E-08 | 96 | 34 |
| Fatty acid biosynthesis | 8.41E-07 | 7 | 18 |
| Axon guidance | 1.16E-06 | 84 | 31 |
| Proteoglycans in cancer | 2.08E-06 | 126 | 34 |
| Pathways in cancer | 8.41E-06 | 234 | 41 |
| TGF-beta signaling pathway | 8.56E-06 | 52 | 30 |
| Endocytosis | 8.56E-06 | 129 | 38 |
| Glutamatergic synapse | 9.06E-06 | 72 | 33 |
| Glioma | 1.11E-05 | 45 | 31 |
| Neurotrophin signaling pathway | 1.11E-05 | 84 | 33 |
| Pancreatic cancer | 1.46E-05 | 48 | 27 |
| Wnt signaling pathway | 1.46E-05 | 91 | 33 |
| Thyroid hormone signaling pathway | 2.14E-05 | 76 | 33 |
| Morphine addiction | 2.41E-05 | 58 | 32 |
| Ras signaling pathway | 2.82E-05 | 137 | 38 |
| mTOR signaling pathway | 4.85E-05 | 46 | 29 |
| Renal cell carcinoma | 4.93E-05 | 49 | 29 |
| ErbB signaling pathway | 5.78E-05 | 62 | 33 |
| Oxytocin signaling pathway | 6.22E-05 | 100 | 31 |
| Prostate cancer | 0.000109167 | 61 | 33 |
| Rap1 signaling pathway | 0.000114454 | 130 | 36 |
| FoxO signaling pathway | 0.000140194 | 85 | 29 |
| Prion diseases | 0.000610539 | 14 | 19 |
| Colorectal cancer | 0.000610539 | 43 | 31 |
| Melanogenesis | 0.001020193 | 64 | 30 |
| Arrhythmogenic right ventricular cardiomyopathy (ARVC) | 0.001260766 | 44 | 30 |
| Estrogen signaling pathway | 0.001357159 | 63 | 29 |
| Retrograde endocannabinoid signaling | 0.00144847 | 63 | 31 |
| Ubiquitin mediated proteolysis | 0.001454394 | 84 | 32 |
| MAPK signaling pathway | 0.001454394 | 146 | 37 |
| Non-small cell lung cancer | 0.001614282 | 37 | 29 |
| Chronic myeloid leukemia | 0.002011181 | 49 | 29 |
| N-Glycan biosynthesis | 0.002454869 | 28 | 25 |
| Sphingolipid signaling pathway | 0.0024629 | 69 | 29 |
| cAMP signaling pathway | 0.0024629 | 118 | 35 |
| Cholinergic synapse | 0.002517567 | 68 | 32 |
| Choline metabolism in cancer | 0.002808964 | 66 | 34 |
| Melanoma | 0.003176705 | 48 | 30 |
| Fatty acid metabolism | 0.003508951 | 25 | 24 |
| Circadian entrainment | 0.004261526 | 63 | 30 |
| Gastric acid secretion | 0.004669872 | 49 | 30 |
| Platelet activation | 0.004705542 | 77 | 32 |
| AMPK signaling pathway | 0.005529721 | 75 | 32 |
| Adrenergic signaling in cardiomyocytes | 0.005613634 | 89 | 32 |
| Regulation of actin cytoskeleton | 0.00632669 | 126 | 36 |
| Long-term potentiation | 0.008072188 | 44 | 31 |
| PI3K-Akt signaling pathway | 0.008072188 | 184 | 38 |
| Hedgehog signaling pathway | 0.008536057 | 35 | 26 |
| Insulin signaling pathway | 0.008907602 | 84 | 34 |
| Adherens junction | 0.009210829 | 51 | 31 |
| Acute myeloid leukemia | 0.013819496 | 38 | 30 |
| Biotin metabolism | 0.016650304 | 2 | 3 |
| Focal adhesion | 0.017971365 | 117 | 35 |
| Type II diabetes mellitus | 0.018319775 | 31 | 24 |
| Prolactin signaling pathway | 0.020508406 | 42 | 30 |
| cGMP-PKG signaling pathway | 0.024289195 | 94 | 32 |
| Glycosaminoglycan biosynthesis - heparan sulfate / heparin | 0.02690339 | 15 | 18 |
| Gap junction | 0.02690339 | 49 | 29 |
| Hepatitis B | 0.038040411 | 79 | 31 |
| Basal cell carcinoma | 0.042559224 | 35 | 27 |
| Circadian rhythm | 0.042606736 | 22 | 22 |
| Aldosterone-regulated sodium reabsorption | 0.042606736 | 26 | 26 |
| p53 signaling pathway | 0.045983486 | 41 | 24 |
| Insulin secretion | 0.046484923 | 51 | 30 |
| Dorso-ventral axis formation | 0.047075025 | 19 | 24 |
| GnRH signaling pathway | 0.047075025 | 54 | 28 |
